# Immunomodulatory Effects Mediated by Dopamine

**DOI:** 10.1155/2016/3160486

**Published:** 2016-10-04

**Authors:** Rodrigo Arreola, Samantha Alvarez-Herrera, Gilberto Pérez-Sánchez, Enrique Becerril-Villanueva, Carlos Cruz-Fuentes, Enrique Octavio Flores-Gutierrez, María Eugenia Garcés-Alvarez, Dora Luz de la Cruz-Aguilera, Emilio Medina-Rivero, Gabriela Hurtado-Alvarado, Saray Quintero-Fabián, Lenin Pavón

**Affiliations:** ^1^Psychiatric Genetics Department, National Institute of Psychiatry “Ramón de la Fuente”, Clinical Research Branch, Calzada México-Xochimilco 101, Colonia San Lorenzo Huipulco, Tlalpan, 14370 Mexico City, Mexico; ^2^Department of Psychoimmunology, National Institute of Psychiatry “Ramón de la Fuente”, Calzada México-Xochimilco 101, Colonia San Lorenzo Huipulco, Tlalpan, 14370 Mexico City, Mexico; ^3^National Institute of Psychiatry “Ramón de la Fuente”, Clinical Research Branch, Calzada México-Xochimilco 101, Colonia San Lorenzo Huipulco, Tlalpan, 14370 Mexico City, Mexico; ^4^Laboratory of Neuroimmunoendocrinology, National Institute of Neurology and Neurosurgery “Manuel Velasco Suárez”, Avenida Insurgentes Sur 3877, La Fama, Tlalpan, 14269 Mexico City, Mexico; ^5^Unidad de Investigación y Desarrollo, Probiomed S.A. de C.V. Cruce de Carreteras Acatzingo-Zumpahuacán S/N, 52400 Tenancingo, MEX, Mexico; ^6^Area of Neurosciences, Department of Biology of Reproduction, CBS, Universidad Autonoma Metropolitana, Unidad Iztapalapa, Avenida San Rafael Atlixco No. 186, Colonia Vicentina, Iztapalapa, 09340 Mexico City, Mexico; ^7^Unidad de Genética de la Nutrición, Instituto de Investigaciones Biomédicas, Universidad Nacional Autónoma de México, Instituto Nacional de Pediatría, Av. del Iman No. 1, Cuarto Piso, 04530 Mexico City, Mexico

## Abstract

Dopamine (DA), a neurotransmitter in the central nervous system (CNS), has modulatory functions at the systemic level. The peripheral and central nervous systems have independent dopaminergic system (DAS) that share mechanisms and molecular machinery. In the past century, experimental evidence has accumulated on the proteins knowledge that is involved in the synthesis, reuptake, and transportation of DA in leukocytes and the differential expression of the D1-*like* (D1R and D5R) and D2-*like* receptors (D2R, D3R, and D4R). The expression of these components depends on the state of cellular activation and the concentration and time of exposure to DA. Receptors that are expressed in leukocytes are linked to signaling pathways that are mediated by changes in cAMP concentration, which in turn triggers changes in phenotype and cellular function. According to the leukocyte lineage, the effects of DA are associated with such processes as respiratory burst, cytokine and antibody secretion, chemotaxis, apoptosis, and cytotoxicity. In clinical conditions such as schizophrenia, Parkinson disease, Tourette syndrome, and multiple sclerosis (MS), there are evident alterations during immune responses in leukocytes, in which changes in DA receptor density have been observed. Several groups have proposed that these findings are useful in establishing clinical status and clinical markers.

## 1. Introduction

Dopamine (DA) is a monoamine that is best known for its neurotransmitter function, and like other neurotransmitters, its effects are not limited to the central nervous system (CNS). Several studies support the notion that DA is a coregulator of the immune system (IS) [1–7], tissues and organs, such as adipose tissue and kidney [8, 9]. Alterations in the DAS have been associated with many health problems, including high blood pressure [10], psychiatric disorders (e.g., schizophrenia), and neurodegenerative diseases (e.g., Parkinson disease).

Based on the involvement of DA in behavioral and cognitive processes, many studies have focused on the nervous system [11–14], describing the general mechanisms, physiological issues, and signaling pathways of the DAS [15, 16]. The existence of DA in the bloodstream suggests the presence of the dopaminergic components that modulate functions in the immune system [17], as in other systems [18]. Studies on monoamines, such as serotonin, DA and its derivatives, and neuropeptides, have become increasingly significant since the 1980s, given their neuroimmunoregulatory functions [19–22].

The CNS and immune system are the main adaptive systems, participating in continuous and functional crosstalk to ensure homeostasis. DA and other catecholamines, such as noradrenaline, function as neuroimmunotransmitters in the sympathetic-adrenergic terminals of the autonomic nervous system, which innervates the primary and secondary lymphoid organs—in addition to the direct local effects that nonsynaptic varicosity secretions have on immune cells [1, 2, 23, 24].

This review focuses on the function of the DAS in the immune system and the function of DA as an immunoregulatory molecule and on the communication between the CNS and IS, based mainly on studies in human cells. We also discuss the clinical aspects of disturbances in the DAS in mental disorders, such as schizophrenia, Parkinson disease, and other clinical conditions that are related to cancer, viral infections, and autoimmunity.

## 2. The Early History of DA and Its Receptors

DA (3-hydroxytyramine; 3,4-dihydroxyphenethylamine; C_8_H_11_NO_2_) was first synthesized in 1910 [25–27]. The initial experiments on DA, in the same year, evaluated its biological effects as a weak sympathomimetic [26, 28]. After nearly 30 years, in 1938, Peter Holtz and colleagues identified L-DOPA decarboxylase in mammals, which uses L-DOPA as a substrate to obtain DA. One year later, Hermann Blaschko in 1939 postulated the biosynthetic pathway of catecholamines, which remains valid and places DA as a precursor of adrenaline and noradrenaline [29].

In subsequent years, observations of small concentrations of DA in several peripheral tissues were reported. Curiously, the name “dopamine” was not adopted until 1952, when a shorter name was proposed by Henry Dale [29]. In the 1950s, the participation of DA in biological processes became recognized, in addition to it being a precursor of adrenaline and noradrenaline, with significant physiological function in the mammalian brain. Arvid Carlsson and colleagues (1957–1959) found that DA has a fundamental function and unique distribution throughout the brain and other tissues [30, 31]. Bertler and Rosengren (Carlsson's students) reported that DA was present in the brains of all of the mammals that they studied but its distribution in the brain differed [32].

This difference, combined with results from other studies that used reserpine, an inhibitor of chromaffin granule amine transporter and synaptic vesicular amine transporter [33, 34], and L-DOPA prompted speculation that DA was involved in the modulation of motor function. Early reports on the distribution of dopamine in animals and humans showed that DA exists primarily in the caudate nucleus in significant amounts [32, 35–37]. At the beginning of the 1960s, the initial studies on Parkinson disease were performed using human tissue from autopsies, demonstrating the absence of DA in the striatum [38].

The idea of providing L-DOPA to patients with Parkinson disease and psychotic disorders arose soon thereafter, leading to the first clinical trials on L-DOPA to mitigate Parkinsonian symptoms [39]. After an extended trial period, L-DOPA was commercialized in 1973 with benserazide, a DOPA decarboxylase inhibitor [40–43].

Moreover, several studies already reported the relevance of DA as a modulator of motor function [45]; the biochemical study of DA receptors (DRs) in the CNS began with Greengard's research, just like the discovery that DA stimulates adenylyl cyclase in the cervical sympathetic ganglia and rat caudate nucleus [46, 47]. The results on stimulation with DA led to the classification of two types of receptors for the second messenger cAMP: stimulatory (alpha-type) and inhibitory (beta-type) [48, 49]. cAMP function depends on the coupling of its receptor (DR) to the heterotrimeric G proteins G*α*-s/olf and G*α*-i/o. The subtypes of receptors are the* D1-like subtype (D1-like)*, which includes D1R and D5R, and the* D2-like subtype (D2-like)*, comprising D2R receptor longer, D2R receptor short, D3R, and D4R [16]. Another important component of the DAS is sodium-dependent dopamine transporter (DAT; gene* SLC6A3*), which was cloned from rat and cow by several groups in 1991 [50–53] and in humans in 1992 [54].

Arvid Carlsson, Paul Greengard, and Eric Kandel were awarded the Nobel Prize in Physiology or Medicine in 2000 for their work on signal transduction in the nervous system [55]. The function of DA as a neurotransmitter precedes its importance in the immune system, based on the many processes in which it participates in the CNS. However, the history of DA and its functions in the IS and other tissues has recently begun.

## 3. Dopaminergic System

The DAS is a vast protein assembly that synthesizes, releases, senses, and metabolizes DA in various cell types in mammals. It also modulates a vast set of neuronal processes. Several examples of brain functions in which DA participates are cognition, motor control, mood, reward systems, pain perception, and sexual behavior [11, 12].

The function of DA outside of the nervous system has only recently been studied. For instance, DA mediates* stem cell-mediated dental repair* with platelets, regulates salt excretion by the kidney, and modulates blood pressure [56, 57]. DA is unable to cross the blood-brain barrier; thus, signaling in the neuronal DAS should be independent of that of the DAS in peripheral systems [11].

### 3.1. DA Concentrations in the Peripheral Region Outside of the CNS

DA in peripheral systems originates from the nervous system and mesenteric region. The concentration of DA in peripheral plasma in humans is approximately 0.1 nmol/L (0.1 pmol/mL) and is derived primarily from sympathetic noradrenergic nerve fibers. This concentration can vary by up to nearly 50-fold for derivatives, such as DA sulfate, after ingestion of a standard meal and according to the region of the circulatory system [58].

The concentration of DA has been assessed in the mesenteric region in samples from abdominal surgeries for gastric adenocarcinoma and pancreatic neoplasm. Abdominal DA concentrations in arterial plasma (samples from the radial and hepatic arteries) and venous plasma (from the right hepatic and portal veins) are approximately 0.312 pmol/mL and 0.937 pmol/mL, respectively (estimated from Figure 1 of Eisenhofer 1997). In addition, by immunoreactivity, tyrosine hydroxylase has been detected in human gastrointestinal mucosa, as have its catalytic activity and the presence of DRs [59, 60]. The concentration of DA and its metabolites in plasma of the portal vein with respect to arterial plasma has demonstrated greater production of DA in the mesenteric organs (12 nmol/min), representing approximately 50% of the DA that is produced in the human body [59].

### 3.2. Dopaminergic System Expressed in Various Tissues and Leukocytes

The physiological mechanisms of the cell signaling and pharmacology of DRs and DA metabolism have been described extensively in the murine CNS [15, 61]. The mechanisms and protein components of this system are likely to be shared between CNS cells and all other tissues in mammals. However, the genes of the DAS in each tissue type are differently expressed differentially, and the protein components must be specifically modulated, based on the function of the cell type. Thus, the genes and proteins of the DAS, such as DRs and DAT, are expressed in a wide range of tissues (e.g., adipose tissue, perivascular adipose tissue, kidney, heart, pituitary, the gastrointestinal tract, and pancreatic beta cells) that regulate processes that differ from those that the CNS governs (e.g., blood pressure regulation, sight process regulation in the retina, vascular permeability regulation in the epithelium, and insulin release) [9, 10, 13, 18, 62–68]. However, many of these studies have been performed primarily in murine models and cell lines (human and murine), potentially creating variations in the results.

The DAS has been observed in murine immune cells [69–73] and human platelets [74]. Recent studies have described its effects on the activation and proliferation of certain cells [75]. The expression of all DRs has been studied in all types of human leukocytes (Table 1). Although their mRNA and protein levels vary between human cell lines, DRs expression is lowest in T lymphocytes and monocytes among all leukocytes, whereas B lymphocyte and NK cell membranes bear the highest levels.

### 3.3. Metabolic Pathways of DA

In the CNS, DA is synthesized through an anabolic pathway that is shared with other catecholamines, such as L-noradrenaline (NE) and adrenaline (E) [61]. Catecholamine metabolism serves as a source of intracellular ROS (*reactive oxygen species*) production, which occurs in Parkinson disease, along with mitochondrial dysfunction [138]. The catecholamine pathway has been proposed to be particularly crucial in the reduction-oxidation (REDOX) homeostasis in cells. It might mediate the overproduction of ROS in neurons, which can compromise the integrity of dopaminergic cells [61]. The effects of DA metabolism in leukocytes, the modulation of the REDOX balance, and the function of DA in mitochondria remain poorly documented [24].

In other tissues, such as the murine kidney, study of the relationship between DRs, oxidative stress, and the REDOX balance has provided information on high blood pressure [10]. The degradation of catecholamines, particularly DA, generates subproducts at low abundance, the concentrations of which depend strongly on the tissue and cell type [61].

A recent mathematical model of DA metabolism in Parkinson disease shows a certain degree of predictability with respect to pharmacological and genetic changes. The authors of this model propose its application* in silico* in the search for molecular approximations that allow the imbalance in DA contents to be restored and changes in oxidative stress to be detected. This model is a preliminary effort, and the authors have commented on future developments and extensions [139]. It would be desirable for such extensions to include peripheral DA systems—for example, studying the metabolism of adrenergic-sympathetic terminals in lymphoid organs and determining their predictive value in human immune system cells.

#### 3.3.1. Anabolism

**Figure 1:**
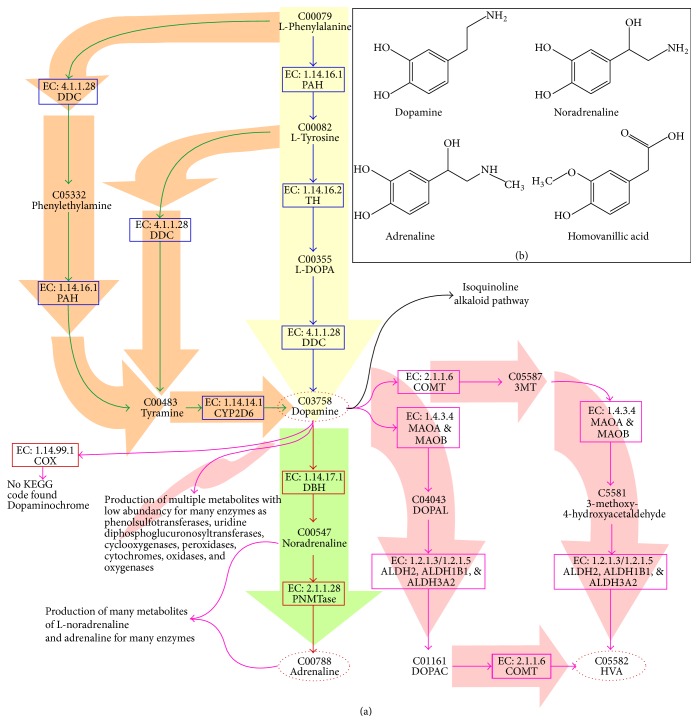
Metabolic pathways associated with DA. The metabolic pathways branching from the catabolism of L-phenylalanine are shown. (a) The dark blue arrows (over light yellow branches) indicate the DA generation pathway, and the red arrows (over light green branches) represent the noradrenaline and adrenaline pathway. Green arrows (over orange branches) show the alternative synthesis pathways to dopamine. The magenta arrows (over pink branches) show dopamine catabolism; the right side shows the normal pathways converging on HVA; the left side shows the catabolic pathways when dopamine concentrations are high in the cytoplasm. Many enzymes can transform dopamine and metabolites, such as COX enzymes (cyclooxygenases), producing dopaminochrome and dopamine quinone [44]. Other enzymes can produce sulfur and glucuronide derivatives. (b) The inset shows the most important products (circled with dotted lines) of metabolic pathways associated with DA. The compounds in these pathways are denoted by Kyoto Encyclopedia of Genes and Genomes (KEGG) code (http://www.genome.jp/kegg/). Enzymes with their classification codes (EC, http://www.chem.qmul.ac.uk/iubmb/enzyme/) and UNIPROT gene names are shown in squares.

The classical pathway of DA synthesis begins with the production of L-tyrosine from L-phenylalanine by phenylalanine 4-hydroxylase (PAH) (EC: 1.14.16.1;* PAH* gene). DA is synthesized in the catecholamine pathway (Figure 1), the first enzymatic step of which is the transformation of L-tyrosine into L-DOPA by tyrosine 3-hydroxylase (TH) (EC: 1.14.16.2;* TH* gene). Both enzymes use molecular oxygen and tetrahydrobiopterin as cofactors of oxidation, rendering them two strongly regulated enzymes. Next, L-DOPA is converted to DA by DOPA decarboxylase (DDC) (EC: 4.1.1.28;* DDC* gene). DA is the precursor of L-noradrenaline, mediated by DA betahydroxylase DBH (EC: 1.14.17.1;* DBH* gene). Finally, L-noradrenaline is converted into adrenaline by phenylethanolamine N-methyltransferase (PNMTase) (EC: 2.1.1.28;* PNMT* gene) [61, 111].

Two alternative pathways of DA synthesis have been identified in the rat brain and human hepatic microsomes. In the first mechanism, DDC transforms L-phenylalanine into phenylethylamine, which in turn is converted into tyramine by PAH; in the second pathway, DDC uses L-tyrosine to produce tyramine. In both cases, tyramine is converted into DA by the cytochrome p-450 CYP2D6 isoform (EC: 1.14.14.1;* CYP2, CYP2D* genes) [140, 141].

The enzymatic activity of TH and PAH in human leukocytes has been detected since the 1980s [112–114]. In addition, the expression and immunodetection of TH in human and mouse leukocytes have been widely reported [77, 79, 83, 106, 107]. On the other hand, DDC expression in human cells has also been documented [79, 109, 110] (Table 1).

In human lymphocytes, the presence and synthesis of certain catecholamines, such as L-DOPA and noradrenaline, the synthesis of which appears to be linked to cholinergic stimulation, have been measured, but they are differentially synthesized between B and T lymphocytes; L-DOPA exists in both cell types, but noradrenaline is only found in T cells [142]. The incubation of human lymphocytes with L-tyrosine and acetylcholine increases L-DOPA and DA levels. In human and murine lymphocytes, incubation with L-tyrosine and L-DOPA increases L-DOPA, DA, and noradrenaline [122, 142, 143].

#### 3.3.2. Catabolism

In neurons, DA is recovered from the* synaptic cleft* by DAT and accumulates in the cytosol, where it is carried to synaptic storage vesicles by synaptic vesicular amine transporter (VAT2). The excess of DA in the neuronal cytosol is degraded by an enzymatic set (Figure 1), comprising monoamine oxidases (MAO-A and MAO-B) (EC: 1.4.3.4; genes* MAOA* and* MAOB*), catechol o-methyltransferase (COMT) (EC: 2.1.1.6; gene* COMT*), aldehyde dehydrogenases (ALDHs) (EC: 1.2.1.3/1.2.1.5; gene type* ALDHs*), alcohol dehydrogenases (ADHs) (EC: 1.1.1.1; gene* ADH*), and aldehyde reductases (ARs) (EC: 1.1.1.21; gene type* AKR*).

Using DA, COMT produces 3-methoxytyramine (3MT), which MAO-A and MAO-B acquire to produce 3-methoxy-4-hydroxyacetaldehyde, which is then used by ALDH to generate* homovanillic acid* (HVA). Alternatively, MAO-A and MAO-B can act on DA to synthesize 3,4-dihydroxyphenylacetaldehyde (DOPAL), which is then transformed into 3,4-dihydroxyphenylacetate (DOPAC) by ALDH. DOPAC is converted to HVA by COMT. DOPAC and HVA are the final principal metabolites of the degradation pathway of DA [61]. However, other subproducts have been observed in the CNS, based on the activity of phenolsulfotransferases (PSTs) and uridine diphosphoglucuronosyltransferases (PAPS), which produce other* derivatives *with sulfate and glucuronic acid, respectively [144–146]. Cyclooxygenases, peroxidases, cytochromes, oxidases, and oxygenases can also oxidize DA. For instance, prostaglandin H synthase (COX) (EC: 1.14.99.1) produces prostaglandin H using DA as a cofactor; subsequently, DA is transformed to generate dopaminochrome. Other examples of low-abundance metabolites that are derived from the catabolism of DA, the functions of which have not been examined, are discussed in Sulzer 1999 and Muñoz 2012 [147, 148].

Other compounds have been detected by spontaneous oxidation of the catechol group of DA and L-DOPA by ROS and amino acids that are derived from cysteine. These compounds are the corresponding quinones, which are associated with oxidative stress, such as DOPA quinone, dopamine quinone, and 6-hydroxydopamine quinone. Beginning with these compounds, a series of oxidation steps with ROS and cysteines occur to generate thioester derivatives and cysteine adducts [44, 61, 147, 149]. These events regarding the catabolism and oxidation of DA and other catecholamines demonstrate that the classical enzymatic degradation pathway of DA must be tightly regulated under regular conditions to produce DOPAC and HVA (waste products). Otherwise, the excess of DA and its metabolites in the cytosol can lead to the formation of cumulative compounds, such neuromelanin, in the lysosomes and cause severe damage to cells. In Parkinson disease, excess production and accumulation of DA and its catabolites in the cytosol can effect mitochondrial dysfunction, oxidative stress, the formation of neurotoxic *α*-synuclein protofibrils, and impairments in protein degradation, which mediate the neurodegeneration of dopaminergic neurons in Parkinson disease [61, 148].

In the mesenteric system (the gastrointestinal tract, spleen, and pancreas), DOPA, DA, and DOPAC are metabolized in the kidneys, plasma, and primarily liver, increasing HVA levels in the portal vein [59]. DOPAC in the bloodstream originates primarily from sympathetic nerve endings and is the precursor of HVA through COMT activity [150].

Generally, the rise in certain metabolites from DA degradation might indicate that cells are damaged by oxidative stress, because when physiological DA concentrations are surpassed, the degradation catabolites (HVA, DOPAL, DOPAC, and 3MT) begin to generate reactive secondary catabolites through spontaneous oxidation by chemical interaction with ROS. Further, when the concentrations of all DA derivatives climb, other enzymes can use them as substrates to generate additional metabolites.

### 3.4. Dopamine Receptors: DRs

Most studies on DRs have been conducted in the CNS, but many reports in other tissues are being published. The human DAS comprises at least six membrane DRs: D1R, D2RL, D2RS, D3R, D4R, and D5R. D2DR has four isoforms that are generated by differential mRNA splicing and have varying functions and sizes. Whereas the two longer isoforms are 443 and 445 amino acids, the short isoforms have 29 and 31 fewer amino acids. The number of DRD3 and DRD4 isoforms has not been determined and is poorly documented in humans and animals [95, 151–155]. In addition, these isoforms have not been linked to any differential functions, unlike DRD2 isoforms.

Human lymphocytes express* DRD5* and two of its pseudogenes, *ψD5DR-1* and *ψD5DR-2*, which are transcriptionally competent and the functions of which are unknown [101, 119, 120]. However, the pseudogenes peptides are likely to generate truncated and nonfunctional receptors [120]. *ψD5DR-1* and *ψD5DR-2* are segmental duplicated chromosomal regions that are 95% identical to DRD5 that cover part of the transcribed region of* DRD5* [120, 121, 156] (Table 2).

Human peripheral blood lymphocytes also express* DRD3* and* DRD4* [95, 99, 101, 151, 152]. Although* DRD1* and* DRD2* were initially believed not to be expressed in these cells [94, 102, 157], recent studies showed that* DRD2* is expressed in T and B lymphocytes, whereas* DRD1* does not appear to be expressed in any leukocyte [88, 97]. By flow cytometry, all DRs, except D1R, are differentially expressed in nearly all human leukocyte cell lineages. The most recent studies show that* DRD1* and D1R are expressed in stimulated T lymphocytes and are linked to the negative regulation of the immune response [76]. In particular, D4R expression is low in all leukocyte subtypes, except NK cells. T lymphocytes and monocytes contain low amounts of all DRs, followed by neutrophils, which primarily express D3R and D5R but at lower levels. B lymphocytes and NK cells have the highest levels of DR [88]. Further, other techniques, such as radioligand binding assay (RBA), have been used to document DR expression in the membranes of immune system cells [94, 158, 159]. D1R, D2R, D3R, and D5R have been observed by immunodetection in mouse bone marrow-derived dendritic cells (BMDCs) [75].

#### 3.4.1. DRs Are G Protein-Coupled Receptors (GPCR)

DRs belong to a superfamily of membrane proteins, called the* G protein-coupled receptor (GPCR) family of class A seven-transmembrane domain receptors (7TM)* [160, 161]. Dopaminergic GPCRs transmit signals toward two transducer-coupled systems: one using heterotrimeric G protein activation and the other using noncanonical G protein-independent, *β*-arrestin-dependent mechanisms. The heterotrimeric G protein complex comprises three subunits (G*α*, G*β*, and G*γ*) that are coupled to the C-terminal end of dopaminergic GPCRs in the inner cell membrane. GPCRs and heterotrimeric G proteins conform two large coupled systems: the DR system and the dopaminergic signal transduction system. The receptor system is formed by a homodimer and sometimes a heterodimer, such as D1R/D2R. Thus, when a signal is received, it is transmitted to the intracellular region through the C-terminus of the receptor, which is coupled to heterotrimeric G protein systems. G proteins initiate signaling cascades by separating the G*α* and G*β*/G*γ* subunits [16, 162].

DRs are functionally classified into the* D1-like* (D1R and D5R stimulatory receptors) and* D2-like* subtypes (D2RL, D2RS, D3R, and D4R inhibitory receptors), based on their ability to stimulate the formation or inhibition of cAMP [48, 49]. The stimulation or inhibition of adenylate cyclases (ACs) (EC: 4.6.1.1) depends on the type of receptor that is coupled to heterotrimeric G proteins. Thus, D1R binds to the G*α*_s/olf_ subunit; D5R binds to G*α*_s_, D2RL, D2RS, or D3R; and D4R binds to G*α*_i/o_. Nevertheless, reports on the putative D1R/D2R heterodimer and D1R and D5R receptors indicate the activation of complexes with the G*α*_q11_ subunit, which acts on phospholipase C (PLC) signaling [16].

The activation of heterotrimeric G proteins is complex, because even if a DR has been classified by AC activation or inhibition, the specific proteins that constitute the heterotrimeric G protein complex are not accurately defined. This is evident when we consider the number of genes that encode for the G*α*, G*β*, and G*γ* subunits. In the human genome, 21 G*α* subunits are encoded by 16 genes, six G*β* subunits are encoded by five genes, and 12 G*γ* subunits are encoded by 12 genes. The variations that arise by* splicing* increase the diversity of heterotrimers [163, 164]. The potential combinations of heterotrimeric G protein complexes suggest a delicate initiation of the signaling mechanism that is coupled to transduction in DR systems. In perspective, further study is needed to determine the specific subunits that form the trimeric complexes that are associated with AC activation or inhibition through the various DRs. These data might help us understand the complex network of interactions that regulate dopaminergic signals in the immune system and its relationship with the CNS.

The complexity of these interactions increases if we also consider that heterotrimeric G proteins are divided into two complementary signaling systems. For D2R in striatal medium spine neurons, the activation of G protein releases the G*α*_i_ subunit (AC activators) and the G*β*/G*γ* subunits, which initiate the PLC activation cascade [165].

The other transducer system that is coupled to DRs is the noncanonical G protein-independent, *β*-arrestin-dependent mechanism, which, although studied less extensively, is just as important. D2R mediates the activation of the multifunctional adapter protein *β*-arrestin 2 (*β*Arr2) with phosphatase A2 (PP2A), which has slower and more persistent effects than the G protein system. Moreover, signals from G protein and *β*Arr2-PP2A have different physiological purposes, demonstrating that the DAS modulates signals by space and time [15, 166]. Further, D2R activation induces a signaling complex that comprises AKT1, PP2A, and *β*-arrestin 2 and downregulates PKA activity [167].

### 3.5. DA Transporter DAT and Synaptic Vesicular Transporters VAT1 and VAT2

The DAS has a plasma membrane-specific DA transporter (*SLC6A3* gene), called sodium-dependent dopamine transporter (DAT). Other transporters, such as chromaffin granule amine transporter (VAT1 protein;* SLC18A1/VMAT1* gene) and synaptic vesicular amine transporter (VAT2 protein;* SLC18A2/VMAT2* gene), participate as well. DAT and VAT2 have not been reported to have functional isoforms, but there are at least two isoforms of VAT1 (*VMAT1* and* VMAT1Δ15*) [117, 118].

VAT1 and VAT2, studied primarily in the brain, are general cytoplasmic amine transporter proteins that reside in the internal vesicular membranes of mammals [34, 168–172].* SLC18A1/VMAT1* is preferentially expressed in neuroendocrine cells, whereas* SLC18A2/VMAT2* is mainly expressed in CNS cells [173].* VAT1Δ15* has been observed in small amounts in the reticulum membrane and is unable to transport serotonin [118]. The function of this protein is yet unknown.

DA is captured and transported to the interior of the cell by DAT, a Na^+^/Cl^−^-dependent DA transporter. Also, SERT (serotonin transporter) is able to take DA to the inside of the cell, though at low rates [115, 173, 174]. In neuroendocrine and endocrine cells, once DA and other monoamines (serotonin, histamine, and norepinephrine) are found in the cytoplasm, they are stored in secretory vesicles by VAT1 and VAT2 through a capture system that is facilitated by a proton gradient that is generated by a vesicular protein, proton ATPase [175–178]. This vesicular confinement modulates monoamine degradation and reuse through a secretory system by exocytosis [61, 173].

DAT and VAT2 transporters are present in the membranes of human peripheral blood lymphocytes [103, 116], and the function and expression of DAT have been verified in leukocytes [69, 91, 104, 105, 179, 180]. However, few studies have examined the expression and function of* SLC18A1/VMAT1* and* SLC18A2/VMAT1 genes* and their protein products, VAT1 and VAT2, in immune cells in rodent models or human cells. This oversight represents an area of interest because mental and mood disorders have been linked to gene polymorphisms [181, 182] and because they might affect the function of the immune system.

Recently, the crystal structure of human DA D3R in complex with a D2R/D3R-specific antagonist, eticlopride, was solved at 2.89 Å resolution and deposited into Protein Data Bank under ID 3PBL [183]. This structure has guided the search for new drugs (agonists and antagonists) against DRs using* in silico* techniques (molecular dynamics simulation and homology modeling) and the creation and redesign of new chemical libraries [184]. The combination of these tools can help discover new molecules with potential use as drugs that are specific and selective for each type of DR [185].

## 4. The Effects of DA on Leukocytes

The immune response is regulated by cytokines, hormones, and neurotransmitters; this regulation is possible because leukocytes have receptors for each one of these soluble factors (Table 3). DA is a neuroregulatory and immunoregulatory molecule that has significant effects on cells that are involved in the immune response. In leukocytes, there is a balance between internal DA, synthesized by DDC, and DA that is transported from blood plasma into the cell through DAT, the latter of which is the primary source of DA in leukocytes [186]. The protein components of the DAS are differentially expressed by leukocyte subtype and the state of cellular activation. One effect of DA is the regulation of leukocytes during activation and function in the immune response. These biological phenomena have recently begun to be examined in various cell types, such as granulocytes, monocytes/macrophages, dendritic cells, and lymphocytes. In this section, we will focus on the evidence showing the relationship of leukocytes with DA and how this catecholamine can regulate leukocyte populations.

### 4.1. Hematopoiesis

Bone marrow (BM) is innervated by autonomic sympathoadrenergic efferent nerve fibers, in which the local microenvironment is critical in the maintenance of hematopoietic stem cells (HSCs). Stem cells are characterized by their capacity for self-renewal throughout the life of an individual and respond to signals that are generated in the microenvironment and identified by cell surface markers, such as CD34 and CD38 [187]. Communication between the CNS and hematopoietic process is known as the “brain-bone-blood triad” and is mediated by many molecules, including such neurotransmitters as DA [188].

Maestroni and colleagues reported the first study on the functions of monoamines in hematopoiesis, performing chemical sympathectomy with 6-hydroxydopamine (6-OHDA) and observing a significantly higher peripheral blood leukocyte count in mice that underwent transplantation with BM [189].

Subsequently, Spiegel et al. demonstrated the expression of D3R and D5R in human CD34^+^ cells by flow cytometry. The more primitive CD34^+^CD38^lo^ cells had higher levels of D3R and D5R. However, D3R and D5R expression was lower in the more differentiated CD3^+^CD38^hi^ cells, and positive correlations existed between DA receptor and an increase in the migration of CD34^+^ cells cord blood that were pretreated with GM-CSF. Their results showed that DA is a chemoattractant that enhances the migration of immature CD34^+^ cells [190]. As discussed, the function of DA in hematopoiesis, mediated by D3R and D5R, might be related to the elevation in circulating CD3^+^ and CD4^+^ lymphocytes, as suggested by the association of polymorphisms in* DRD1* and* DRD5* with these cell counts [191].

Additional work of the function of DA demonstrated the amelioration of neutropenia and the restoration of the number of colony-forming unit-granulocyte macrophage (CFU-GM) colonies in the bone marrow of mice that were treated with 5-fluorouracil (5FU). These results are consistent with reports that have indicated that DA can be used safely as an antiangiogenic drug for malignant tumors [192] (see Section 5).

### 4.2. Granulocytes

Granulocytes are fundamental immune cells, based on their abundance and rapid activation in the presence of foreign elements. Granulocytes contain granules in their cytoplasm that harbor various inflammatory and antimicrobial mediators that effect their defensive activities. Depending on the content of their granules, these cells are classified in eosinophils, basophils, and neutrophils [193].

Eosinophil function in immunity is related to the response against parasites [193]. These cells have a higher density of D3R and D5R receptors and a low density of D2R and D4R, whereas D1R is not detected [88]. No study has reported the effects of DA on eosinophils. Similarly, it is unknown whether basophils, involved in the allergic response, express DRs or respond to DA.

Neutrophils are the most abundant leukocyte population and have a significant function at the beginning of an inflammatory response [193]. These cells contain intracellular catecholamines, such as DA, epinephrine (E), and norepinephrine (NE), and several of their metabolites, such as DOPAC, 3MT, HVA, DL-3,4-dihydroxyphenylglycol (DHPG), and metanephrine (MET). Further, neutrophils synthesize and degrade such amines.* In vitro*, incubation with *α*-methyl-*p*-tyrosine, an inhibitor of TH, reduces the intracellular concentration of DA, NE, and its metabolite, DHPG. Similarly, reserpine, a VAT inhibitor, lowers intracellular concentrations of DA and NE, and desipramine, an inhibitor of NE transporter (NET), decreases intracellular NE concentrations. These findings implicate the existence of catecholamine storage and catecholamine reuptake mechanisms in neutrophils [194].

Neutrophils express D3R and D5R DA receptors and, at lower densities, D2R and D4R [88], which allows DA to modulate neutrophil function. Neutrophils (from peripheral blood) that are incubated with DA reduce their phagocytic activity dose-dependently, just like the production of oxygen reactive species and chemotaxis, with the maximum effect at 100 ng/mL [195]. Further, at 10 *μ*Mol/L and 100 *μ*Mol/L, DA influences the viability of neutrophils from healthy volunteers and patients with Systemic Inflammatory Response Syndrome (SIRS) inducing apoptosis after 12 h of incubation in healthy volunteers and 6 h in SIRS patients, whereas respiratory burst activity remains undisturbed [196].

DA also reduces the density of the adhesion molecules CD11b (Mac-1) and CD18 in neutrophils, decreasing endothelial adhesion. Even in neutrophils that have been stimulated with LPS or TNF-*α*, DA (10 nM and 100 nM) slows transendothelial migration and impedes chemoattraction by IL-8 versus cells that are stimulated in the absence of DA [197]. In that regard, neutrophils that are activated with FMLP (N-formyl-methionyl-leucyl-phenylalanine) and incubated with pharmacological concentrations of DA (261 nM) increase their density of CD62l (L-selectin) and decrease the density of CD11b. In the same report, DA at physiological concentrations (0.26 nM) had no effect on CD62I or CD11b [198].

### 4.3. Monocytes/Macrophages

Monocytes are found in peripheral blood, and on entering tissues, they complete their differentiation into macrophages. Macrophages have high phagocytic capacity toward microorganisms and dead cells, secrete large amounts of cytokines, and present antigen in the context of MHC II [193]. According to McKeena and colleagues, human monocytes bear a higher density of D2R and D3R compared with D4R and D5R [88]. There are few reports about the effects of DA on monocytes; resting peripheral blood CD14^+^ monocytes express* DRD4* but not other DA receptors [86]. Also, human CD14^+^ monocytes from peripheral blood and the U937 cell line (premonocytes) harbor intracellular DA, E, and NE, and CD14^+^ monocytes also contain some metabolites, such as 3MT, DHPG, and MET [199].

DA modulates the phenotype and function of monocytes/macrophages. An* in vitro* study in chicken macrophages demonstrated that high DA concentrations (1–5 *μ*g/mL) are cytotoxic, causing up to 53% of cells to die. Incubation with DA at 0.1 and 0.5 *μ*g/mL for 1 hour improved their phagocytic activity, but extended exposure to DA (3 h) lowered this activity, although the percentage of Fc receptor-positive macrophages increased due to DA [200]. Haskó and colleagues demonstrated in LPS-activated peritoneal macrophages of Swiss mice that D2R stimulation using agonists (bromocriptine and quinpirole) and antagonists (sulpiride) lowered TNF-*α* and nitric oxide (NO) secretion. In contrast, D1R stimulation with the antagonist SCH23390 only downregulated NO production compared with controls [201].

In an elegant study, Gómez and colleagues reported that macrophages from guinea pigs that were immunized* in vivo* for 7 days using DA agonists (bromocriptine, leuprolide, and pergolide) or DA antagonists (chlorpromazine, SCH23390, metoclopramide, sulpiride, veralipride, alizapride, and cisapride) and primary macrophage cultures from guinea pigs that were stimulated* in vitro* with these drugs had increased Fc*γ* receptors expression. The DA agonists improved the clearance of IgG-sensitized RBCs (*in vitro* recognition of IgG-sensitized RBCs by splenic macrophages) and enhanced the membrane expression of Fc*γ* receptors in macrophages; in contrast, the DA antagonists impaired macrophage Fc*γ* receptor expression. Thus, the disturbance in Fc*γ* receptor expression is more extensive when D1R is stimulated and less so on activation of D2R [202].

Bergquist and colleagues examined whether the binding of NF-*κ*B to DNA was inhibited by DA in nontransformed human peripheral blood monocytes and transformed human monocyte cell lines that were activated with LPS (10 ng/mL). Cell proliferation declined at 10 *μ*M DA in peripheral blood monocytes and at 100 *μ*M in the monocyte cell line after 24 h; however, the low concentrations (1 *μ*M and 0.1 *μ*M) had no effects. They also observed that DA suppressed LPS-mediated activation of NF-*κ*B and LPS-induced binding of NF-*κ*B to the TNF-*α* promoter dose-dependently—an effect that might be attributed to the inhibition of NF-*κ*B translocation from the cytoplasm to nucleus by DA [203]. In 2002, Haskó and colleagues showed that DA has anti-inflammatory effects by binding to its receptors and through other mechanisms. Using the J774.1 cell line and C57B1/6 mice peritoneal macrophages that were stimulated with LPS (10 *μ*g/mL) and DA (0.01 *μ*M–100 *μ*M), they observed that IL-12 p40 secretion and mRNA decreased dose-dependently, whereas IL-10 secretion was increased. These effects were not caused by DR stimulation (addition of the DR antagonists SCH23390 and raclopride did not inhibit the effects) but by the stimulation of *β*-adrenergic receptors as determined by the addition of the *β*-adrenergic antagonist propranolol, which had partial inhibitory activity [204].

Human monocyte-derived macrophages (HMDMs) from healthy donors express the* SLC6A3/DAT, SLC18A2*/*VMAT2, TH, DDC*,* DRD1 [79–81], DRD2, DRD3, DRD4*, and* DRD5* genes and the D1R, D2R, D3R, D4R, DAT, VAT2, TH, and DDC proteins on the cell surface and in the cytoplasm, suggesting that these cells contain the machinery for synthesis, reuptake, and response to DA [79]. Another report showed that DA modulates cytokine secretion in HMDMs with and without LPS stimulation; LPS-activated HMDMs that were stimulated with elevated concentrations of DA (2 *μ*M and 20 *μ*M) increased IL-6, CCL2, CXCL8, and IL-10 secretion while TNF-*α* secretion declined. Conversely, lower DA concentrations (20 nM and 200 nM) affected only TNF-*α*, IL-6, and CCL2 secretion and upregulated IL-10, albeit insignificantly. In cells without LPS stimulation, 2 *μ*M and 20 *μ*M DA enhanced IL-6 and CCL2 secretion. These findings suggest that macrophages develop differential responses, depending on the microenvironment (inflammatory or homeostatic), that are modulated by DA [79].

#### 4.3.1. DA Receptor Roll in HIV Infection in Macrophages

DA also has effects on macrophages with regard to D2R-mediated HIV replication. This effect has been observed in HMDMs of healthy donors that have been infected* in vitro* with the HIV_ADA_ and HIV_YU2_ strains [80] and in Jurkat cells (T-cell-derived line) that have been transfected with the HIV proviral genome—an effect that is mediated by the activation of NF-*κ*B [205]. D2R activation by the agonist quinpirole stimulates ERK1 by phosphorylation and increases HIV replication dose-dependently compared with unstimulated infected cells and cells stimulated with the D1R agonist SKF82958 [80].

In macrophages, the entry of HIV via CD4 and CCR5 in the plasma membrane depends on gp120 binding [206], and an increase in the density of CCR5 and CD4 enhances HIV infectivity [207]. Incubation of HMDMs with IL-4 and IL-10 upregulates CCR5 and CD4, accelerating the infection [207, 208]. Similarly, a recent study showed that DA facilitates HIV entry through CCR5 and that TAK779, a CCR5 inhibitor, impedes viral entry [209]. These data suggest that entry of the virus requires the activation of DRs and is inhibited by a global DR antagonist, such as flupenthixol, through effects that do not depend on viral concentration.

The average percentage of infected HMDMs rises with high concentrations of DA—between 10^−5^ and 10^−8^ M—dose-dependently, with a “*steep threshold*” at approximately 10^−8^ M. This finding confirms that CCR5 and DA-mediated DRs activation are necessary for viral entry in HMDMs. Specifically,* D1-like* and* D2-like* are the receptors that are activated and involved in HIV entry, indicating that there is a common pathway of activation that depends on Ca^2+^ mobilization [209]. Methamphetamine enables infection in HMDMs and increases the activity of viral reverse transcriptase and CCR5 density, thus downregulating IFN-*α* and STAT1 protein expression. STAT1 is a signal transducer and transcriptional activator that mediates cellular responses to interferons, cytokines, and growth factors. However, the D1R antagonists SCH23390 and SKF83566c block HIV_Bal_ ineffectiveness [81]. These data suggest the participation of dopamine receptors during macrophage infection by HIV which might have clinical applicability but demands more clinical trials.

### 4.4. Dendritic Cells

Dendritic cells (DCs) are the most efficient antigen-presenting cells of the immune system, with key functions in the induction of adaptive immune responses, immune tolerance, and the modulation of immune responses [193].* Human monocyte-derived dendritic cells* (Mo-DCs) express the* D1-like* and* D2-like* receptors in the membrane, of which the* D2*-*like* receptors predominate functionally [123, 124]. DCs synthesize DA and store it in vesicles near the cell membrane, as observed in Mo-DCs, in which DA synthesis and storage increase when intracellular cAMP levels rise. Further, Mo-DCs liberate DA during their interaction with naive CD4^+^ T lymphocytes, which promotes polarization toward the Th2 phenotype; antagonism of Mo-DCs* D2-like* receptors with sulpiride and nemonapride raises cAMP levels, releasing DA and influencing naive CD4^+^ T lymphocytes, for example, by increasing the Th2/Th1 ratio (through the IL-5 : IFN-*γ* relationship), upregulating CCR4 (a Th2-type receptor), and decreasing CXCR3 (a Th1-type receptor) expression [123].

DA also has effects on murine bone marrow-derived dendritic cells (BMDCs), which express the molecular components that are needed to respond to, synthesize, store, and degrade DA. BMDCs bear D1R, D2R, D3R, and D5R on the membrane and vary their expression profile according to their state of activation: mature (stimulated by LPS) and immature (without stimulus). In the mature state, the intracellular enzyme TH, low levels of* Scl18a2/Vmat2* mRNA,* Slc6a4/Sert*,* maoa*, and* maob* have been observed without detectable* Slc6a3/dat*,* Slc6a2/Net1* (NE transporter), or* Dbh* mRNA. The same results have been obtained for immature cells, but there was more* Slc18a2/Vmat2* mRNA [75]. Although BMDCs do not express DAT in the membrane or have quantifiable mRNA levels, SERT is present, and this transporter can carry DA at a low velocity [174].

In BMDCs, D5R appears to participate in maturation and regulate signaling pathways and cytokine release, thus contributing to the activation and proliferation of CD4^+^ T lymphocytes. BMDC activation with LPS significantly decreases the density of D5R in the membrane. On stimulation with SKF38393, a selective D1R/D2R agonist, the phosphorylation of ERK1/2 increases. Notably, D5R is linked to IL-12 and IL-23 production; it has been observed that mature* Drd5* knockout (Drd5KO) cells express less* Il-23a* (IL-23) and secrete less IL-12. These reports implicate an autocrine regulatory mechanism during cell maturation in which the release of DA and D5R activation selectively promote the secretion of certain regulatory cytokines. Similarly, CD4^+^ T lymphocytes significantly decrease IL-2 secretion and proliferation when they are activated and incubated using Drd5KO cells versus WT cells. This effect is not observed in CD8^+^ T lymphocytes, suggesting that D5R expression in DCs facilitates the strong priming of CD4^+^ T lymphocytes [75].

### 4.5. Lymphocytes

Lymphocytes are primordial cells of the adaptive immune response that recognize antigens in their molecular context. Depending on their ligands, lymphocytes have many subpopulations with a wide variety of functions; these cells can modulate, regulate, and coordinate the activities of other leukocyte populations through cytokine secretion and at the same time, lymphocytes can respond to circulatory levels of cytokines, hormones, and neurotransmitters [193].

#### 4.5.1. Synthesis of Dopamine and Receptors

In the late 20th century, lymphocytes were demonstrated to have the metabolic ability to synthesize catecholamines and their metabolites; they also release and recapture these molecules, responding to them by expressing catecholamine receptors. The initial reports on DA and DOPAC in lymphocytes were based on cerebrospinal fluid and human T and B lymphocyte cultures; these studies reported that intracellular catecholamine concentrations and the inhibition of TH with *α*-methyl-*p*-tyrosine downregulated intracellular catecholamines, which rose on exposure of the cells to DA [20]. Later, the presence of DA, L-DOPA, and NE in lymphocytes was confirmed by electrospray ionization mass spectrometry [210].

Another study reported that human peripheral lymphocytes exhibit intracellular DA, L-DOPA, and NE at concentrations that are detectable by HPLC and that T lymphocytes only contain L-DOPA and NE.* In vitro*, T lymphocytes that have been incubated with L-tyrosine (5 × 10^−5^ M) or L-DOPA (10^−8 ^M–10^−5 ^M) increase their intracellular NE concentrations. This finding suggests the presence of catecholamine synthesis pathways in these cells [143]. Marino and colleagues noted that PBMCs contain DA, NE, E, and metabolites, such as DOPAC, 3MT, HVA, DHPG, MET, and VMA. This group reported that the addition of *α*-methyl-*p*-tyrosine decreased DA and NE concentrations intracellularly and in the medium; further, incubation with desipramine or GBR12909, 2 catecholamine reuptake inhibitors, significantly increases DA and NE levels in the medium, indicating the existence of an active recapture mechanism [116].

In the late 1990s, a study in rats confirmed that lymph node, splenic, and thymic lymphocytes contain intracellular catecholamines (DA, NE, and E), observing* TH* mRNA and protein in these cells. Also, the authors found that ConA-activated (concanavalin A) (5 *μ*g/mL) lymphocytes had higher intracellular catecholamine and TH concentrations than unstimulated cells [108, 211]. CD4^+^ CD25^−^ Teff lymphocytes and CD4^+^ CD25^+^ Treg were proven to express TH and intracellular L-DOPA, DA, E, and NE and some catecholamines metabolites. Further, incubation of CD4^+^ CD25^+^ Treg with reserpine (an inhibitor of VAT1) downregulated intracellular catecholamine concentrations, whereas concentrations in the medium rose [83].

The first report on DAT in peripheral blood lymphocytes was performed using several techniques. By radiobinding assay (RBA) using a specific ligand for DAT, [^3^H]-GBR12935, it demonstrated specific binding in the membrane of human lymphocytes. By western blot, DAT, VAT1, and VAT2 were expressed, whereas DA and VAT2 showed immunoreactivity in cytoplasmic areas, corresponding to vacuoles, by immunofluorescence. Finally, DAT and VAT1 were detected in the plasma membrane and cytoplasm, respectively [103]. Later, Marazziti and colleagues confirmed DAT expression by RBA using [^3^H]-WIN35428 in human lymphocytes, 1 of the most DAT-selective radioligands. This group observed [^3^H]-DA reuptake, suggesting the presence of a DA reuptake mechanism [179]. In addition, Ferrari and colleagues reported that DA modulates its synthesis in human T and B lymphocytes from peripheral blood through PKC activation using 12-O-tetradecanoylphorbol-13-acetate (TPA) at 100 ng/mL. This activity increases* TH* mRNA levels and intracellular catecholamines—effects that are significantly inhibited with DA (1 *μ*M) and SKF30393, a D1-*like* agonist, demonstrating that the stimulation of D1-*like* receptors impedes catecholamine synthesis [77].

The search for DA receptors in lymphocyte has been a difficult task. Unstimulated human lymphocytes express D2R, D3R, D4R, and D5R, but the activation of these cells modifies the expression of the receptors. In 1991, the initial data on specific dopamine binding sites in human lymphocytes were obtained using [^3^H]-DA, the binding of which declines considerably in the presence of such substances as cocaine and other inhibitors of biogenic amine uptake [69]. Subsequently, D5R was detected in the membranes of human lymphocytes by RBA using the dopaminergic antagonist [^3^H]-SCH23390, as were 3* DRD5* mRNA sequences (by RT-PCR) and the transcription of 2 pseudogenes [101]. Similarly, D3R expression was observed in the membrane using the specific ligand [^3^H]-7-hydroxy-N,N-di-*n*-propyl-2-aminotetralin ([^3^H]-7-OH-DPAT) [95, 212], as were its full-length mRNA sequence and a shorter variant transcript, generated by alternative splicing [95, 151].

Another study with [^3^H]-sulpiride in human lymphocytes by RBA characterized the D2-*like* receptors in the membrane and their similarities with D2R and D4R and, to a lesser extent, D3R [158]. Conversely, the expression of D1-*like* receptors was described in 2 studies by RBA using [^3^H]-SCH23390 [73, 102] and immunocytochemistry [102], in which the expression of D5R, but not D1R, was demonstrated. Other studies on D4R in human lymphocytes detected it by RT-PCR [99, 100] and by RBA using [^3^H]-clozapine [159] and western blot [100].

In 1998, Ricci and colleagues measured the expression in membrane-bound D2-*like* receptors by RBA and immunocytochemistry with greater precision. They observed that the ligand [^3^H]-7-OH-DPAT is not specific for D3R, because, on incubation with anti-D3R and anti-D4R, the interaction decreases 53% and 32%, respectively, whereas anti-D2R has no effect [96]. Another study measured D3R and D4R by RBA using the radioligands [^3^H]-7-OH-DPAT, [^3^H]-spiperone, and [^3^H]-nemonapride and immunocytochemistry [94]. Cosentino and colleagues showed that intracellular catecholamines fall when TH is inhibited with *α*-methyl-*p*-tyrosine and reserpine in primary human PBMC cultures; reserpine also decreases catecholamine concentrations in the medium. This study also demonstrated that cell lines from hematopoietic precursors, such as NALM-B (pre-B), Jurkat (T lymphoblastoid), and U937 (promonocytic), synthesize catecholamines, because their intracellular DA, E, and NE decrease on incubation with *α*-methyl-*p*-tyrosine and reserpine [199].

In 2002, McKenna et al. performed flow cytometry to confirm the membrane expression of the three D2-*like* receptors and D5R and the absence of D1R [88]. The existence of D5R in the membrane was later confirmed by RBA using the radioligand [^3^H]-SCH23390. By RT-PCR, expression of* DRD2*,* DRD3*,* DRD4*, and* DRD5*—but not* DRD1—*was observed [93]. DA receptors and DAT, with the exception of D4R, were detected in B lymphocytes and in several malignant B cell lines; also, this report showed higher* DRD1* and* DRD2* transcript levels versus* DRD3* and* DRD4* [78]. In 2014, by 5-color flow cytometry, Kustrumovic and colleagues showed that naive T CD4^+^ T lymphocytes, central memory CD4^+^ T lymphocytes (TCM), and effector memory CD4^+^ T lymphocytes (TEM) expressed D1R, D2R, D3R, D4R, and D5R in the membrane at various densities between subpopulations. In CD4^+^ T lymphocytes that were stimulated with anti-CD3/CD28 (0.01–0.1 *μ*g/mL) for 48 hours, the density of the five DA receptors in the membrane was altered after activation, with increases in the D1-*like* (71% to 84%) and D2-*like* (55% to 97%) receptors. With regard to the levels of DRs, the frequency of these receptors is higher in apoptotic cells than in viable T lymphocytes. However, the stimulation of viable T lymphocytes increases receptor density to similar levels as in apoptotic lymphocytes [85].

#### 4.5.2. Effects of Dopamine in Lymphocytes

The immunomodulatory effects of DA have significant relevance in understanding the relationship between the immune system and CNS. Reports in rodent and human lymphocytes have found that DA receptors in PBMCs are functional and activate signaling cascades that change the phenotype and function of lymphocytes. Some groups have reported the effects of DA on cytokine secretion, cell adhesion, and chemotaxis in human and rodent lymphocytes.

In 2001, Levite and colleagues suggested the importance of DA in integrin-mediated cellular trafficking and extravasation of human T lymphocytes in the brain and periphery, based on findings that 7-hydroxy-DPAT (a D3R agonist), bromocriptine, and pergolide (D2R agonists) activate T lymphocytes, upregulating *β*-integrins expression (mainly *α*4*β*1 and *α*5*β*1) and increasing adhesion to fibronectin (FN) [213]. In 2004, Cosentino and colleagues showed that, at various doses, DA has opposing effects on oxidative metabolism and apoptosis in human lymphocytes. At low doses (0.1–5 *μ*M), DA decreases the concentration of reactive oxygen species (ROS) and inhibits apoptosis through stimulation of D1-*like* receptors. However, at high concentrations (100–500 *μ*M), DA increases intracellular ROS levels and lymphocyte apoptosis [24]. In 2005, Besser and colleagues demonstrated that human resting T lymphocytes express D2R, D3R, and D5R on their membrane and that their stimulation upregulates the secretion and expression of cytokines, such as TNF-*α* and IL-10. Stimulation with DA between 10^−4 ^M and 10^−7 ^M increased TNF-*α* secretion through D3R and D1-*like* receptors stimulation, whereas IL-10 secretion was mediated by D2R and D1-*like* receptors stimulation; in contrast, concentrations of DA between 10^−9^ M and 10^−14^ M did not have any discernible effects [90].

Watanabe and colleagues showed that CD8^+^ T lymphocytes selectively express D3R and that its stimulation mediates chemotaxis and CD8^+^ T lymphocyte adhesion. DA and its agonist, 7-OH-DPAT (100 nM), increase CD45RA^+^ CD8^+^ naive T lymphocyte chemotaxis, whereas the combination of DA and other chemokines enhances chemotaxis in CD45RA^+^ CD4^+^ and CD45RA^+^ CD8^+^ T lymphocytes. Stimulation with only CCL19 (10 nM), CCL21 (10 nM), and CXCL12 (0.1 nM) significantly induces the migration of CD4^+^ and CD8^+^ lymphocytes, but the addition of DA (1 nM) induces the selective migration of CD45RA^+^CD8^+^ T lymphocytes. In addition, D3R stimulation induces the adhesion of CD45RA^+^ CD8^+^ T and CD45RO^+^ CD8^+^ T lymphocytes to FN [86]. Conversely, Strell and colleagues showed that human CD8^+^ T lymphocytes expressed D3R and D4R and, to a lower extent, D5R. They also found that these receptors were downregulated on cellular activation, with the exception of D5R. Stimulation with DA (1 *μ*M) decreased the activation of these cells by anti-CD3/CD28, decreasing the expression and secretion of IL-2 by reducing ERK1/ERK2 phosphorylation. This stimulation also increased I*κ*B, which lowers NF-*κ*B phosphorylation levels. This effect prevents the creation of an autocrine IL-2 loop, which is needed for optimal activation of T lymphocytes [98].

With regard to animal models, Kipnis and colleagues showed that DA regulates the adhesion and chemotaxis of mouse Treg lymphocytes by stimulating D1-*like* receptors via ERK. CTLA-4 expression decreases in Treg lymphocytes that are incubated with DA or SKF38393, as does IL-10 secretion at concentrations of 10^−5 ^M. These effects are attributed to ERK phosphorylation. Finally, this group found that DA affects the adhesion and chemotaxis of Treg lymphocytes; adhesion to SPG (extracellular matrix proteins that are associated with injured tissues) decreases dose-dependently (10^−9^–10^−5 ^M), and DA downregulates the receptor for CCR-4, affecting the migration of these cells toward macrophage-derived chemokines (MDCs) [84].

Watanabe and colleagues showed by* in vivo* assays that intraperitoneal administration of DA or 7-OH-DPAT to mice selectively attracts CD44^low^ CD8^+^ T lymphocytes, effecting their accumulation in the peritoneal cavity. They also reported that DA mediates the homing of naive CD8^+^ T lymphocytes toward secondary lymphoid tissues through D3R, because U99194A, a D3R antagonist, reduces the number of CD44^low^ CD8^+^ T lymphocytes in the inguinal lymph nodes. Similarly,* in vitro* stimulation of L1.2 pre-B lymphocytes with DA or 7-OH-DPAT (100 nM) increases calcium efflux and elicits a selective chemotactic response for CD44^low^ CD8^+^ T lymphocytes [86].

Other reports have shown that DA modulates the activation, proliferation, and differentiation of lymphocytes in humans and rodents. In human cells, Bergquist and colleagues showed that ConA-activated peripheral blood lymphocytes stimulated* in vitro* with DA (10 *μ*M and 100 *μ*M) slow their proliferation, differentiation, and synthesis of IFN-*γ* dose-dependently, with complete inhibition reached at 500 *μ*M. Further, incubation with DA from 10 *μ*M to 500 *μ*M completely inhibits the production of antibodies in pokeweed mitogen- (PWM-) stimulated B lymphocytes [20]. This group also reported that human lymphocytes (activated with ConA or PWM) stimulated with DA (10 *μ*M and 100 *μ*M) produce less IL-4 but experience 2.8-fold greater apoptosis; apoptotic markers, such as Bcl/Bax and Fas/FasL, are also upregulated [125].

In 2001, Saha and colleagues demonstrated that high DA concentrations in serum affect the proliferation of CD4^+^ and CD8^+^ T lymphocytes and the cytotoxicity of lymphocyte activated killer T cells (LAK-Ts) from patients with lung carcinoma and healthy subjects. The patients had high DA concentrations in plasma compared with healthy subjects (48.6 ± 5.1 pg/mL and 10.2 ± 0.9 pg/mL, resp.).* In vitro*, this high concentration (48 pg/mL) slowed the proliferation of CD4^+^ and CD8^+^ T lymphocytes from patients and healthy donors, which did not occur at physiological concentrations (10 pg/mL). Similarly, LAK-Ts from patients and healthy subjects had less cytotoxic activity, attributed to D1R stimulation due to the rise in intracellular cAMP [214]. Further, the group performed the same experiments in CD4^+^, CD8^+^, and cytotoxic T lymphocytes from patients with uncoping stress and healthy subjects, showing that these patients also had high plasma DA concentrations (46.6 ± 3.9 pg/mL) versus the latter (10 ± 2.7 pg/mL). The effect of high DA concentration was the same as in patients with lung carcinoma [215].

Ghosh and colleagues reported that DA also affects TCR-mediated signaling in T lymphocytes. Incubation with DA at 3–5 ng/mL for 1 to 3 days inhibited the proliferation and secretion of IL-2, IFN-*γ*, and IL-4 in anti-CD3-activated T lymphocytes, due to D2R and D3R stimulation. They also showed that DA downregulates the nonreceptor tyrosine kinases Lck and Fyn, which are important in TCR signaling; this effect can decrease lymphocyte activation and cytokine secretion [89]. In 2006, Sarkar and colleagues observed that D4R stimulation in human T lymphocytes induces quiescence by upregulating lung Krüppel-like factor 2 through inhibition of ERK1/ERK2 phosphorylation. T lymphocytes that were activated with anti-CD3/CD28 and a D4R-specific agonist (PD168,077 or APT724 at 1 *μ*M) experienced less proliferation, which was not observed in unstimulated T lymphocytes. Similarly, cells that were activated for 24 hours with PD168,077 (1 *μ*M) had equal levels of IL-2 secretion and CD69 and CD25 expression as in nonactivated lymphocytes. PD168,077 also prevented a decline in KFL2 expression, a transcription factor that regulates quiescence and inhibition of ERK1/ERK2 phosphorylation [100].

Studies in rodent cells* in vivo* and* in vitro* have demonstrated the effects of DA on lymphocyte activation, proliferation, and differentiation, such as Tsao et al., who observed* in vivo* that DA governs splenocyte proliferation, based on the intravenous administration of agonists for D1-*like* (SKF38393) and D2R (LY171555) (1, 5, and 10 *μ*g/Kg) in BALB/cByJ mice. They showed that agonists enhance LPS- or ConA-induced splenocyte proliferation; however, intraperitoneal administration of the neurotoxin MPTP (20 *μ*g/kg) reduced endogenous DA levels and suppressed proliferation [216]. Carr and colleagues determined that chronic administration of L-DOPA (126 mg/Kg every 5 days) or L-DOPA combined with domperidone, an agonist of D2R, affects proliferation and cytokine secretion in mouse splenocytes. This group demonstrated that L-DOPA increases the proliferation of spleen cells that have been stimulated with ConA or anti-CD3 by approximately 2.2-fold [217].

In 2011, a study reported that stimulation of D1-*like* receptors suppresses ovalbumin antigen-induced neutrophilic airway inflammation in OVA TCR-transgenic DO11.10 mice. These mice were nebulized with OVA or LPS and received a D1-*like* antagonist (SCH23390) by oral administration before OVA administration. SCH23390 significantly inhibited OVA-induced neutrophilic airway inflammation, due primarily to its ability to halt the infiltration of neutrophils, macrophages, and lymphocytes. IL-17 and IL-22 synthesis and infiltration of Th17 cells in the lung were also lower. Conversely, IL-23 production was suppressed in DC11c APCs in response to LPS/anti-CD40 [218].

*In vitro*, Cook-Mills and colleagues demonstrated that DA (10 *μ*M and 100 *μ*M) and NE inhibit the activation of splenic lymphocytes from BALB/c mice. However, this effect is not blocked by adrenergic or dopaminergic antagonists, suggesting that the inhibitory effect is mediated by other lymphocyte receptors [219], such as serotonin [7], hormone, and cytokine receptors [193]. According to Josefsson and colleagues, mouse splenocytes produce catecholamines and are stimulated by DA, L-DOPA, and NE, which are enhanced by L-tyrosine and inhibited by TH inhibitors. L-DOPA and DA (0–500 *μ*M) dose-dependently suppress mitogen-induced proliferation and differentiation of mouse splenocytes, even with short treatment times. Moreover, L-DOPA and DA (500 *μ*M) also inhibit IL-2, IL-6, and IFN-*γ* synthesis and IgG and IgM secretion [122].

Bergquist and colleagues reported that, at high concentrations (100–500 *μ*M), DA inhibits the proliferation of T lymphocytes and the secretion of IL-2, IL-6, and IFN-*γ* at 500 nM [125]. Tsao et al. demonstrated that DA promotes the proliferation of splenocytes from BALB/cByJ mice in response to LPS or ConA [216]. Carr and colleagues showed that mouse splenocytes (BALB/c) that were treated with L-DOPA secreted less IFN-*γ* but produced the same amount of IL-4 [217]. Huang and colleagues found that T lymphocytes from mouse mesenteric lymph nodes, which express the five DA receptors, are polarized toward the Th2 phenotype when D2-*like* receptors are stimulated, suggesting that this effect involves the cAMP-CREB pathway. In the same lymphocytes, stimulation with the D1-*like* agonist SFK38393 reduced only IFN-*γ* secretion; in contrast, quinpirole, a D2-*like* agonist, enhanced IFN-*γ* and IL-4 secretion and decreased proliferation and cAMP and phosphorylated CREB content [87].

DA also has indirect effects on the phenotype and function of lymphocytes. This catecholamine first modulates the function of its target cell, which in turn affects the function and phenotype of the lymphocyte with which it interacts; these effects on human cells have been described by other groups.

In 2007, Cosentino and colleagues demonstrated that DA is released by Treg, allowing them to regulate their activity. However, when Treg do not release DA, they experience autoregulation and lose the ability to suppress Teff proliferation. CD4^+^CD25^−^ Teff and CD4^+^CD25^+^ Treg from healthy donors express the five DA receptors. Treg have detectable mRNA levels of* SCL18A1*/*VMAT1*,* SCL18A2*/*VMAT2*,* DRD2*,* DRD3*,* DRD4,* and* DRD5*, whereas Teff only express* SCL18A1*/*VMAT1* and* SCL18A2*/*VMAT2*. When DA release is inhibited by reserpine (1 *μ*M) in Treg/Teff cultures, Treg diminish their mRNA levels and secretion of IL-10 and TGF-*β* through stimulation of D1-*like* receptors. These events significantly reduce the proliferation of CD3/CD28-activated T lymphocytes but do not affect the secretion of TNF-*α* and IFN-*γ* [83]. Nakano and colleagues demonstrated that D2-*like* antagonists induce the differentiation of Th17 cells (*in vitro*), mediated by DCs, using a mixed lymphocyte reaction (MLR) between* human monocyte-derived dendritic cells* (Mo-DCs) and naive CD4^+^ T lymphocytes. These antagonists increased the secretion of IL-17 in 16-hour cultures of Mo-DCs and CD4^+^ T lymphocytes that were activated with anti-CD3/CD28; in contrast, D1-*like* antagonists (SCH23390, SKF83566, and LE300) decreased IL-17 levels [124].

A 2004 study by Kipnis et al. in mice showed that Treg from splenic lymph nodes had lower regulatory activity on stimulation of D1-*like* receptors through ERK activation. Moreover, the incubation of Treg/Teff with DA (10^−7 ^M) increased Teff proliferation 2-fold, which was also observed in T lymphocytes that were activated with anti-CD3 and IL-2. This group also found that the negative regulatory activity of Treg on Teff proliferation is inhibited by genistein, a MEK and ERK inhibitor. This inhibition was also observed with the ERK inhibitor PD98059 [84].

Mori and colleagues reported that D1-*like* receptors mediate immediate and late-phase skin reactions by promoting Th2 differentiation and mast cell degranulation. In* in vivo* Th1-type contact hypersensitivity and Th2-type atopic dermatitis models, they observed that SCH23390 does not affect Th1-type contact hypersensitivity but suppresses immediate-type (ITRs) and late-phase reactions (LPRs) in the atopic dermatitis model. In addition, SCH23390-treated mice had higher* IFNG* and lower* IL-2* mRNA levels in the ear skin versus untreated mice. This report also used bone marrow-derived mast cells (BMMCs), fetal skin-derived cultured mast cells (FSMCs), and naive Th2 splenic lymphocytes as* in vitro* models. Using these models, the group demonstrated that mast cells and CD4^+^ T lymphocytes have D1-*like* receptors and that DA increases mast cell degranulation and Th2 cell differentiation; both of these activities were abrogated by SCH23390. In T lymphocytes, the ratio of* IL-4/IFNG* mRNA rose on addition of DA. Also, DA increased the release of *β*-hexosaminidase from BMMCs dose-dependently; this effect was also observed when D1-*like* receptors were stimulated through IgE-triggered Fc*ε*R1 [220].

#### 4.5.3. Other Approaches

Ilani and colleagues hypothesized that dopaminergic activation of blasts (cells that cross the blood-brain barrier) induces the Th1 phenotype and effects changes in membrane surface markers. The authors suggest that these alterations are transferred from blasts to peripheral resting T lymphocytes by neurotransmitter-mediated brain regulation of peripheral T lymphocytes. In this work, blast formation was induced from peripheral blood lymphocytes of healthy donors (cells that have been activated with mitogen and IL-2 that express VLA-4 on their membrane); these blasts were incubated with the D2R/D3R agonist quinpirole (10^−5 ^M–10^−7 ^M) for 8 hours. Quinpirole downregulates* IL-4* and* IL-10* but increases* IFNG* expression but has no effects on resting T lymphocytes.

Further, this group noted differential responses between CD4^+^ and CD8^+^ blasts. CD4^+^ blasts had lower* IL-4* and* IL-10* mRNA levels, but* IFNG* mRNA rose. In contrast, CD8^+^ blasts only upregulated* IFNG* mRNA. Differences were also noted in adhesion molecules between T lymphocytes and blasts—unchanged in the former during quinpirole stimulation but with the latter upregulating* IL-2RA* (CD25) mRNA and decreasing* CXCR3* levels.

In resting T lymphocytes, incubation with blast supernatant for 24 hours without quinpirole increased* IL-4* and* IL-10* mRNA levels, whereas that with quinpirole upregulated only* IFNG*. These findings support the hypothesis that blasts that are stimulated with DA in the CNS trigger the phenotype implantation in peripheral blood resting T lymphocytes [97].

### 4.6. NK Cells

NK cells constitute less than 10% of all circulating lymphocytes and have significant functions in the immune response against viruses, intracellular bacteria and in tumor cell destruction [193]. Studies on the effects of DA on NK cells were initially performed in rodents and demonstrated that DA has antitumor effects against* Ehrlich ascites carcinoma* cells [221]; in Swiss mice with transplantable Ehrlich ascites carcinoma, cancer cells are controlled through increased splenic NK cells [222]. Other studies in NK cells from the spleens of APO-SUS rats with a hyperdopaminergic phenotype [223] and mice with the* slc6a3/Dat knockout* phenotype [126] reported a decline in NK cell activity and dampened* mitogen-induced cytokine responses*. Another study has reported that mouse splenic NK cells express the 5 DA receptors in their membrane and that the stimulation of D1-*like* receptors with SKF38393 increases the density of D1R and D5R; SKF38393 also improves the cytotoxic response against YAC-1 lymphoma cells (Moloney leukemia virus-induced mouse lymphoma) through cAMP-PKA-CREB signaling, but stimulation of D2-*like* receptors with quinpirole impairs NK lymphocyte function [82].

There are differences in the phenotype and function of human and murine NK cells—human NK cells express membrane D2R, D3R, D4R, and D5R but lack D1R [88, 92]. Without activation, human NK cells have no disturbances in phenotype or function when they are exposed to DA; however, stimulation with high concentrations of rIL-2 (≈200 IU) for 5 days induces the overexpression of D5R. At concentrations between 10^−9 ^M and 10^−18^ M, DA reduces NK cell division and inhibits IFN-*α* secretion dose-dependently, wherein D5R signaling is compromised [92].

## 5. Clinical Implications of the Effects on the Dopaminergic System

Central and peripheral DA can directly or indirectly regulate the immune system in several pathological conditions, such as neurodegenerative (Parkinson disease, Alzheimer disease, and Lesch-Nyhan syndrome), psychiatric (schizophrenia), and immune diseases (multiple sclerosis, encephalomyelitis, and rheumatoid arthritis, among others), and conditions that have an addictive component, such as alcoholism.

In terms of a physiopathological perspective, disturbances in the levels of central DA affect the function of lymphocytes, because DA is supplied by the sympathetic nervous system to primary and secondary lymphoid tissues, modulating a wide range of immune activities, such as the regulation of innate immune and adaptive responses [224]. Several studies have reported disturbances in central DA production under pathological conditions, the most common of which is Parkinson disease, characterized by the selective destruction of dopaminergic neurons in the substantia nigra. However, recent studies indicate that DA production and the expression of dopaminergic receptors are dysregulated in other neurodegenerative and autoimmune diseases, which significantly impacts the immune response.

The expression of dopaminergic receptors in lymphocytes from patients with neurodegenerative and autoimmune diseases has recently been proposed as a diagnostic biomarker and a marker of pathological severity, because the variations in the density of DA receptors on lymphocytes are usually similar to what is observed in the brain. In this section, we catalog the evidence on variations in the expression of DRs on lymphocytes in various pathologies (Table 4).

### 5.1. Neurodegenerative Diseases

Changes in activation of the immune response in patients and in experimental models of neurodegenerative diseases have been described and implicated in their pathogenesis. Abnormalities in the number and function of circulating lymphocytes are linked to an increase in the production of proinflammatory mediators. The evidence in this section strongly suggests that the DAS participates in the modulation of the immune response.

#### 5.1.1. Parkinson Disease

Parkinson disease is a neurodegenerative pathology that is characterized by the dysfunction and degeneration of dopaminergic neurons in the substantia nigra, neuroinflammation, and motor disturbances. In animal models in which the selective loss of dopaminergic neurons from the substantia nigra is induced by systematic administration of methyl-4-phenyl-1,2,3,6 tetrahydropyridine (MPTP), significant changes in the immune response have been observed. For example, proinflammatory cytokines, such as IFN-*γ*, IL-2, IL-17, and IL-22, are upregulated in the spleen and mesenteric lymphatic ganglia; T-bet, a fundamental transcription factor in Th1 differentiation, is downregulated in T cells; and the expression of Foxp3, a transcription factor that induces the development and maintains the function of Treg, rises [225].

Regulation by the central DAS modifies peripheral immune functions. For instance, MPTP-induced depletion of DA in the striatum promotes tumor growth, which is associated with dysfunctional cytotoxic activity in T lymphocytes and NK cells [2, 226]. In this regard, NK cell activity wanes on injury to the nucleus accumbens in animal models [227].

Some studies propose that the activation of circulating lymphocytes is able to regulate the neurodegenerative events in the substantia nigra in Parkinson disease. The stimulation of D3R in CD4^+^ T lymphocytes decreases their synthesis of IL-4 and IL-10 while promoting IFN-*γ* production. Thus, D3R is a relevant target in the physiopathology of Parkinson disease. DRD3-deficient mice that have been treated with MPTP are susceptible to neurodegenerative events in the substantia nigra after receiving CD4^+^ T lymphocytes from MPTP-treated wild-type mice [127].

Similarly,* Drd3*KO mice are resistant to MPTP-induced neurodegeneration but become susceptible on transfer of CD4^+^ T lymphocytes from MPTP-treated wild-type mice. However, they are not prone to MPTP-induced neurodegeneration when they receive CD4^+^ T lymphocytes from D3R-deficient mice [127].* DRD3* expression in lymphocytes is reduced in Parkinson disease patients and correlates with disease severity, possibly due to changes in D3R density in other lymphocyte populations. These data show that D3R in T lymphocytes favors the activation and acquisition of the Th1 phenotype, suggesting that D3R in CD4^+^ T lymphocytes has an important function in the physiopathology of the murine model of Parkinson disease [127].

#### 5.1.2. Other Neurodegenerative Diseases

Patients with a likely diagnosis of Alzheimer disease have a low density of D2-*like* receptors on lymphocytes. This decrease is also observed in postmortem samples of brains from Alzheimer disease patients [228]. However, Cosentino and colleagues did not observe any differences in the mRNA levels of DRs in lymphocytes in patients with a probable diagnosis of Alzheimer disease [229]. These controversial results might be attributed to the statistical parameters, such as sample size (number of participants), age, gender, and the presumptive diagnosis. With regard to the DAS, the lymphocytes of patients with probable Alzheimer disease experience an increase in the immunoreactivity of dopamine *β*-hydroxylase [230]; nevertheless, more studies are needed to determine the function and effects of DA on lymphocytes in the physiopathology of Alzheimer disease.

Studies have reported changes in the expression of DRs in lymphocytes in various pathologies of the CNS. Lesch-Nyhan syndrome is a neurogenetic disorder that is caused by the complete deficiency of hypoxanthine-guanine phosphoribosyltransferase, which effects severe motor disturbances, predominantly dystonia (numbness) and occasionally chorea (involuntary movements); these secondary symptoms are related to disturbances in the production of DA in the CNS. Lesch-Nyhan syndrome patients have higher levels of* DRD5* in lymphocytes, rendering it a potential biomarker for the diagnosis of this disease and prompting the use of L-DOPA in Lesch-Nyhan patients as an alternative treatment [128].

### 5.2. Psychiatric Disorders

#### 5.2.1. Schizophrenia

Studies on disturbances in the DAS in schizophrenia patients have reported changes in the expression of DA receptors. In the 1980s, a study examined the binding of [^3^H]-spiperone, a specific dopaminergic antagonist, to peripheral blood lymphocytes from healthy volunteers, 27 patients with acute schizophrenia under no treatment, and 16 psychiatric patients as a control group by RBA. The study did not find any differences in binding parameters between the healthy and psychiatric control groups, whereas the binding of [^3^H]-spiperone increased significantly in lymphocytes from schizophrenia patients with a slight decrease in affinity [70].

At the beginning of the 21st century, Kwak and colleagues performed an 8-week longitudinal study to measure D3R and D5R expression in peripheral blood lymphocytes from 44 patients who had been treated pharmacologically for over three years, 15 drug-naive schizophrenic patients, 28 drug-free patients, and healthy controls.* DRD3* mRNA in drug-naive patients climbed significantly compared with medicated patients and healthy controls, and* DRD5* mRNA was considerably higher only versus medicated patients. In drug-free and drug-naive patients, the expression of the receptors rose two weeks after antipsychotic treatment was begun, decreasing at 8 weeks of treatment. When the drug-naive and drug-free patients were divided into two groups by* DRD3* expression before the treatment, those with higher* DRD3* levels presented with more severe psychiatric symptoms [231]. In the same year, Ilani and colleagues observed a significant increase in* DRD3*, but not* DRD4,* mRNA in peripheral blood lymphocytes from 14 schizophrenic patients who were not under medication with respect to healthy subjects. This rise was not affected by treatment with typical or atypical antipsychotic drugs. Based on these data, the group implicated* DRD3* as an identification and tracking marker [232].

In 2006, Boneberg and colleagues measured DA receptor (D1R–D4R) expression in neutrophils, monocytes, B lymphocytes, NK cells, and CD4^+^ and CD8^+^ lymphocytes from 10 schizophrenic patients, reporting a significant increase in* DRD3* mRNA in T lymphocytes and downregulation of* DRD4* mRNA in T CD4^+^ lymphocytes compared with healthy volunteers [233]. In contrast, Vogel and colleagues reported a decrease in* DRD3* mRNA in peripheral blood leukocytes from 13 schizophrenic patients and 11 patients with bipolar disorder, subdivided as follows: drug-naive (never having ingested antipsychotics), drug-free (without any treatment for at least four weeks), and drug-treated (under pharmacological treatment). The schizophrenic drug-naive and drug-free patients had significantly less* DRD3* mRNA compared with healthy subjects. However, patients who were under treatment had higher* DRD3* mRNA levels during the six weeks of treatment; consequently, these levels were similar to those in healthy controls [234].

Another study attempted to identify schizophrenia markers in peripheral blood lymphocytes from 13 drug-naive/drug-free patients. In a microarray analysis,* DRD2* and inwardly rectifying potassium channel (Kir2.3) were overexpressed compared with healthy subjects; this effect was confirmed, based on the elevated mRNA levels of both genes. The group suggested using these genes to predict schizophrenia [235]. Urhan-Kucuk and colleagues studied 55 schizophrenia patients and 51 healthy subjects to determine whether* DRD3* expression in peripheral blood lymphocytes could be used as a marker of disease. They noted no significant difference in* DRD3* mRNA levels between schizophrenic patients and healthy subjects. However, across schizophrenia subtypes (residual, disorganized, and paranoid), these levels differed between the disorganized and paranoid subtypes and between disorganized schizophrenia and healthy subjects, prompting the authors to conclude that* DRD3* mRNA could be used as a peripheral marker of schizophrenia subtype [236].

Recently, Brito-Melo and colleagues used flow cytometry to measure membrane expression of D2R, D4R, and serotonin receptors in CD4^+^ and CD8^+^ peripheral blood T lymphocytes from schizophrenic patients who had been treated pharmacologically for ten years. They correlated these levels with several clinimetric scales: Brief Psychiatric Rating (BPRS), Positive and Negative Syndrome (PANSS), and Involuntary Movement (AIMS). The group observed significant overexpression of D4R in CD8^+^ and CD4^+^ T lymphocytes from schizophrenic patients and upregulation of D2R in CD8^+^ T lymphocytes; in contrast, D2R levels were lower in CD4^+^ T lymphocytes. Further, BPRS and PANSS scores correlated with CD8^+^D2R^+^ lymphocyte levels, and AIMS scores were positively associated with CD4^+^D2R^+^ T lymphocytes levels and inversely related to CD4^+^D4R^+^ T levels [131].

In 2013, Liu and colleagues measured* DRD2* and* SLC6A3/DAT* expression in peripheral blood leukocytes from 25 patients with acute schizophrenia, 27 patients with chronic schizophrenia, and healthy subjects to determine whether their mRNA levels correlated with PANSS scores. There was no significant difference in* DRD2*, but* SLC6A3/DAT* was higher in patients with chronic schizophrenia compared with healthy subjects. In addition, they noted a correlation between* DRD2* mRNA levels and positive scores on the PANSS—but only in acute schizophrenia patients [91].

Another study analyzed the* DRD3*,* DRD2*, and* DARPP-32* (dopamine and cyclic adenosine 3′,5′-monophosphate-regulated phosphoprotein-32) mRNA levels in peripheral blood lymphocytes from healthy subjects, patients with an unspecified psychotic disorder, and patients with schizophrenia/schizophreniform disorder, examining the relationship between these genes and the psychopathological state of the patients. The study demonstrated that* DRD3* mRNA in T lymphocytes differed considerably between the three groups but that* DRD2* and* DARPP-32* levels were similar. Further,* DRD3* expression correlated with the excitement factor on the PANSS in patients with schizophrenia/schizophreniform disorder. According to the authors,* DRD3* mRNA levels can be used as a diagnostic marker to differentiate patients with early psychosis from healthy controls [237].

### 5.3. Autoimmune Diseases

#### 5.3.1. Multiple Sclerosis

Multiple sclerosis (MS) is the most common immune-mediated demyelinating disease of the CNS. This condition causes disability in 2.3 million people worldwide. In MS, myelin-reactive CD4^+^ Th lymphocytes enter the CNS, where they interact with resident cells, promoting inflammation, demyelination, and neurodegeneration [238]. Th cell subsets that are involved in the pathogenesis of MS include Th1 cells, which secrete the proinflammatory cytokines TNF-*α* and IFN-*γ*, and Th17 lymphocytes, which produce IL-17 [239].

The typical treatment for MS is IFN-*β*, which induces the production of DA and other catecholamines in human lymphocyte cultures [240]. DA downregulates IL-17 and IFN-*γ* production by PBMCs in patients with relapsing-remitting MS and healthy controls, strengthening the evidence of the potential benefit of dopaminergic agents in MS [3].

In untreated patients, the expression and activity of D1-*like* receptors (but perhaps not D2-*like* receptors) in circulating PBMCs are tempered [238], although untreated MS patients express less* DRD5* mRNA and protein without an increase in D3R [129, 130]. Immunomodulatory drugs, such as IFN-*β*, restore the functional responsiveness of DRs on lymphocytes. Moreover, IFN-*β* therapy appears to shift the balance of DRs in lymphocytes from predominantly D2-*like* in the cells of untreated patients toward primarily D1-*like*. D1-*like* receptors mediate most of the dopamine-dependent inhibition of human T lymphocyte proliferation and cytotoxicity, whereas D2-*like* receptors induce T lymphocyte proliferation and adhesion. Upregulation of D1-*like* receptors is thus expected to be beneficial in MS [129, 130, 238, 241].

Functional dysregulation of Treg contributes to disease pathogenesis and activity in autoimmune mouse models of the CNS and in patients with MS. Thus, the use of DR agonists in MS might suppress Treg via D1-*like* receptors, with detrimental effects [238]. Notably, treatment with IFN-*β* downregulates D1-*like* receptors on Treg and impedes the ability of DA to inhibit Treg function [3].

These findings suggest that the dopaminergic pathways in circulating lymphocytes have relevant immunomodulatory functions in the pathology of MS, impacting the development of drugs for patients with MS—DR agonists have beneficial effects as an add-on to immunomodulatory treatments with such agents as IFN-*β*, and they might act preferentially on D1-*like* rather than D2-*like* receptors [238].

#### 5.3.2. Encephalomyelitis

Experimental autoimmune encephalomyelitis (EAE) is an experimental model of human MS. Balkowiec-iskta and colleagues showed that injury to the dopaminergic system modulates the clinical course and inflammatory reaction during EAE; this group studied the effects of dopamine depletion with 1-methyl-4-phenyl-1,2,3,4-tetrahydropyridine (MPTP) in C57BL mice with EAE (induced by the MOG 35–55 peptide). They found that MPTP decreased striatal DA levels, and the mean number of inflammatory cells in the spinal cord infiltrate was significantly higher in MPTP + MOG 35–55-treated versus MOG 35–55-treated mice. The mortality rate in mice with a dysfunctional dopaminergic system was lower than in MOG 35–55-treated mice. Also,* Il1b* mRNA was significantly upregulated in the MPTP + MOG 35–55 group, correlating with clinical progression of the disease; this IL-1*β* increase could be responsible for these changes, causing a more severe course of EAE [242].

Nakano et al. showed that antagonizing D1-*like* receptors suppresses IL-17 production and prevents EAE in SJL/J mice—animals that were treated with L750667 (a D2-*like* antagonist) developed hyperacute EAE, the progression of which was accelerated and quickly resulted in death. Conversely, mice that were administered SCH23390 (a D1-*like* antagonist) did not present with any clinical symptoms. Also, splenocytes from EAE mice that were treated with SCH23390 for 30 days produced less IL-17, whereas IFN-*γ* levels rose. The transfection of BMDCs from SJL/J mice that were treated with antagonists* in vitro* into SJL/J mice affected the same clinical incidence of EAE. These results suggest that D1-*like* receptor antagonists ameliorate EAE, an effect that is accompanied by an increase in IFN-*γ* and the suppression of IL-17 in antigen-specific T lymphocytes [124]. In 2012, Prado and colleagues demonstrated in C57BL/6 mice with EAE* in vivo* that D5R-deficient DCs that were transferred prophylactically into wild-type mice mitigated the severity of EAE. Further, mice into which D5R-deficient DCs were transferred experienced a significant reduction in the percentage of Th17 cells that infiltrated the CNS compared with animals that received wild-type DCs, whereas the percentage of Th1 lymphocytes remained in similar levels [75].

#### 5.3.3. Rheumatoid Arthritis

Rheumatoid arthritis (RA) is a chronic inflammatory disease that is characterized by pannus tissue, consisting of synovial fibroblasts (SFs), macrophages, and lymphocytes. The inflammatory milieu in the joint activates resident SFs and transforms them [243]; in particular, SFs increase their expression of D1R and D5R [244]. RA has a high predominance of Th1 and Th17 lymphocytes; further, DA localizes to DCs in the synovial tissue of RA patients and significantly increases its levels in this fluid [244]. In RA patients, DCs release DA during antigen presentation to naive CD4^+^ T lymphocytes [245], which raises IL-6-dependent IL-17 production via D1-*like* receptors, in response to T lymphocyte activation by anti-CD3 and anti-CD28 [244].

The involvement of D2R in RA has been demonstrated in several murine models. Its activation mitigates clinical symptoms, and* Drd2* knockout (*Drd2*KO) mice develop severe symptoms of RA; D2R antagonists induce the accumulation of IL-17^+^ and IL-6^+^ T cells in synovial fluid, exacerbating the inflammatory process. Similarly, RA patients express low levels of* DRD2* in lymphocytes, which is linked to disease severity [132]. Based on this evidence, D2R agonists have been proposed to be therapeutic agents for RA.

D5R levels in B lymphocytes from RA and osteoarthritis patients are lower than in healthy volunteers, whereas those of D2R and D3R are higher [133]; Nakano and colleagues have suggested that DA that is released by DCs activates the IL-6-Th17 axis, aggravating synovial inflammation in RA [246]. Thus, clinical protocols have been developed in which clinical researchers should use D2R agonists, such as bromocriptine and cabergoline, to lower prolactin synthesis and secretion by infiltrating synovial fibroblasts and lymphocytes in patients with RA—the improvement in RA activity might be attributed to a significant decrease in the secretion of prolactin by immune cells [247, 248], although these results are not conclusive.

#### 5.3.4. Systemic Lupus Erythematosus

Systemic lupus erythematosus is an autoimmune disease that is characterized by the dysfunction of several organs, including the liver and brain, due to a dysregulated immune system. In PBMCs of systemic lupus erythematosus patients,* DRD2* is downregulated, whereas* DRD4* increases compared with a control group. The decrease in D2R levels might be associated with the reduction in the function and numbers of Treg cells in this pathology [134].

### 5.4. Miscellaneous Clinical Implications

#### 5.4.1. Glomerulonephritis

Glomerulonephritis encompasses a range of immune-mediated disorders that cause inflammation in the glomerulus and other compartments of the kidney [249]. DA mediates the control of renal sodium excretion, and DRs have been detected in various regions of the nephron—it has been reported that DA is synthesized in the renal proximal tubules. A defect in renal DA receptor function and DA production has been suggested to accelerate the pathogenesis of hypertension [250]. Conversely, in a brain-dead model in rats, the administration of DA reduced monocyte infiltration in renal tissue, indicating that DA has a direct anti-inflammatory effect that is mediated by D1-*like* and D2-*like* receptors stimulation [251].

In another study by Hoeger et al. using the same brain-dead model in rats, the changes in cytokine and chemokine expression were measured to determine the mechanism by which DA lowers renal inflammation. This study evaluated the expression of IL-6, IL-10, macrophage chemoattractant protein 1 (MCP-1), and cytokine-induced neutrophil chemoattractant 1 (CINC-1), a rat homolog of IL-8. No significant changes were observed in IL-6, IL-10, and MCP-1, but CINC-1 was significantly downregulated in the brain-dead animal group that was treated with DA compared with controls, implicating this change as an anti-inflammatory mechanism that is induced by DA during renal inflammation [252]. Kapper et al. also demonstrated that DA dose-dependently inhibits the production of the chemokines Gro-*α*, ENA-78, and IL-8 in proximal tubular epithelial cells (PTECs) [253]. Collectively, these findings support the function of DA as an immunomodulator in glomerulonephritis.

#### 5.4.2. Cancer and Angiogenesis

The dopaminergic system has garnered significant interest in angiogenesis and tumor immunity. Endothelial cells express components of the dopaminergic system; thus, DA governs angiogenesis, prompting an examination of the molecular mechanisms that are associated with modulation of tumor immunity, its mechanisms of control, and the link between tumor immunity and angiogenesis [214, 254, 255].

In a murine model, the antitumor effects of DA on Ehrlich ascites carcinoma cells already have been reported [221]. This inhibitory effect suppresses the growth of cancer cells by increasing the number of peripheral large granular lymphocytes (LGLs) and the activity of NK cells. Even in healthy mice (without Ehrlich cells) that have been treated with DA, these effects are also observed [222].

The low incidence of certain types of cancer in schizophrenic patients, in contrast with the high incidence in patients with Parkinson disease, reflects the inhibitory effects of DA on cancer cell growth. This hypothesis is based on the finding that schizophrenic patients express a hyperdopaminergic system, whereas Parkinson patients are hypodopaminergic. However, it is unknown if disruptions in the dopaminergic system in the CNS contribute to the development of tumor angiogenesis outside of the CNS [256–260]. However, the effects of DA on schizophrenic patients might be linked to medication with DR antagonists, suggesting that the development of specific cancers is DR-dependent [261].

Conversely, DA selectively inhibits vascular permeability and the angiogenic activity of VEGF. This inhibitory effect is mediated by the activation of D2R and the induction of the endocytosis of VEGFR-2 [262, 263]. Further, at low pharmacological doses, DA delays tumor angiogenesis by inhibiting VEGFR-2 phosphorylation in endothelial cells that express D2R, as reported in rat malignant gastric tumors (adenocarcinoma type) and xenotransplanted human gastric cancers in mice. In malignant tumors of the stomach from humans and rats, endogenous DA and TH enzyme are absent compared with normal tissue [264].

Another study demonstrated that DA acts through D2R to inhibit the proliferation of gastric cancer cells that have been induced by insulin-like growth factor receptor-I (IGF-IR). This inhibition is mediated by the upregulation of Krüppel-like factor 4 (KLF4) [265]. Also, the ablation of peripheral dopaminergic nerves increases angiogenesis, density, and microvascular permeability, permitting the growth of malignant tumors in mice [266]. Recent reports have demonstrated that the administration of DA stabilizes and normalizes tumor blood vessels by acting on pericytes and endothelial cells, primarily by activation of D2R [254, 267]. Thus, DA has been proposed to be a safe antiangiogenic drug for the control of tumor progression [192].

The relationship between DA, transformed cells, and the immune system is unknown, but it is likely that a link exists with mechanisms of the identification and elimination of abnormal cells, because DA inhibits tumor angiogenesis and stimulates tumor immunity; nevertheless, more studies are needed to examine this issue.

#### 5.4.3. Diabetes Mellitus

The involvement and significance of DA as a neurotransmitter and immunomodulator have been studied, but its effects on glucose homeostasis and pancreatic *β*-cell function are unknown [268]. Diabetes mellitus is a group of metabolic diseases that are characterized by hyperglycemia, resulting from defects in insulin secretion, insulin activity, or both. Chronic hyperglycemia in diabetes is associated with long-term damage, dysfunction, and failure of organs, especially the eyes, kidneys, nerve fibers, heart, and blood vessels [269].

DA and its derivatives can act directly in pancreas; the networks between the CNS and pancreatic islets are based on the central vagal connection through the parahypothalamic ventricular nucleus [270]. The pancreas expresses DA receptors, depending on cell type; mRNA of the five DA receptors is expressed in *β*-cells [271], but only D1R, D2R, and D4R proteins have been detected in these cells [271–273].

Insulin production depends on the concentration of DA [274]. In humans, neuroleptic drugs cause hyperinsulinemia in normal subjects and are associated with diabetes in psychiatric patients [268]. DA and its agonists modulate *β*-cell activity, but it is unknown whether DA promotes or inhibits insulin secretion [275]. The activation of D2R receptor inhibits glucose-stimulated insulin secretion in isolated islets from rodents and *β*-cell lines [271], and another study in which D2R was knocked out in the INS-1 832/13 mammalian cell line reported an increase in insulin secretion [276]. García-Tornadú et al., using a global D2R knockout mouse, showed that the disruption in D2R impairs insulin secretion and causes glucose intolerance [277].

One regulatory mechanism that explains the effects of DA states that DA in pancreatic *β*-cells involves a dopaminergic negative feedback loop that regulates insulin secretion from human and murine pancreatic islets. According to this model, DA is stored in *β*-cells and is cosecreted with insulin; this endogenous DA acts in an autocrine/paracrine manner on insulin-secreting *β*-cells that express D2-*like* receptors [67, 278].

The effects of DA on the immune system in this disease are not well elucidated. One study showed that the D1-*like* antagonist SCH-23390 has a preventive effect on diabetes mellitus that occurs naturally in NOD mice. In this* in vivo* model, Th17 lymphocytes mediate the development of diabetes in NOD mice. The islet infiltrates appear to be composed of mononuclear cells that are positive for IL-23R (a specific Th17 marker), but SCH-23390 significantly decreases the levels of infiltrating cells. This group reported that the antagonism of D1-*like* receptors suppresses IL-17 production and prevents naturally occurring diabetes in NOD mice, accompanied by an increase in IFN-*γ* production [279].

Diabetes mellitus is a disease with an important inflammatory component; understanding the regulation of DA in the immune system and the significance of the inflammatory condition in the development of this disease will require a more detailed examination of the regulation of DA in this condition with regard to immunity.

### 5.5. Addictions

The function of the D1R and D2R receptors in the brain is linked to the reward system and might be affected by extended drug use. Several reports indicate that the expression of DRs in immune system cells is altered during addiction. For instance, in patients who suffer from alcohol dependence,* DRD1* mRNA levels in lymphocytes are higher compared with healthy controls [136]. Other studies have demonstrated that in alcohol dependence syndrome patients, DRD4 is upregulated in peripheral lymphocytes versus a control group [135].

Similarly, opioid addicts experience an increase in* DRD3* expression in circulating lymphocytes compared with healthy controls [280]. On the other hand, a chronic rise in D1R might be a risk factor that predisposes individuals to some type of addiction allowing one to diagnose the severity of the addictive condition [281]. The expression of DA receptors in the immune system might be a valuable biomarker of the risk for pathologies and addictive conducts; thus, it has also been measured in computer game addicts, in whom* DRD5* levels in circulating lymphocytes are lower than in control subjects [137]. Notably, the personality of volunteers has been correlated with the expression of the* DRD3* and* DRD4* receptors [282]. These findings suggest that DA receptor expression in the periphery constitutes a significant link between neurobiological processes and immune system function.

## 6. Conclusions

Based on the activity of DA as a neurotransmitter, studies on the DAS have focused primarily on the CNS. Recently, however, copious experimental evidence indicates that the DAS has important physiological functions in the immune system. The human DAS is complex and comprises many elements; leukocytes have frequently been demonstrated to synthesize, release, perform reuptake of, and metabolize DA. In certain cases, as in Treg cells, DA is released to elicit autocrine effects, implicating the existence of a peripheral DAS that is independent of the CNS.

In this review, we have discussed the immunomodulatory effects of DA by activating DRs, which are differentially expressed in leukocytes, depending on cell type, activation state, DA concentration, and duration of exposure to DA. These effects regulate many cellular processes, such as cell activation, cell adhesion, proliferation, respiratory burst, chemotaxis, apoptosis, cytotoxicity, and cytokine and antibody secretion, and several changes at the phenotypic level and function in certain cell types. All of this activity is linked to the intracellular concentration of cAMP and the activation status of second messengers and transcription factors.

The information that we have here is based on existing reports, but the effects of DA on the immune system require further characterization. The peripheral DAS is dysregulated in patients with psychiatric disorders, neurodegenerative diseases, and other conditions, such as multiple sclerosis. However, more research is needed to demonstrate that peripheral disturbances in the DAS are equivalent to those in the CNS, which will facilitate the identification and characterization of new peripheral biomarkers for diagnostic purposes and the evaluation of the therapeutic efficacy of pharmacological treatments in these illnesses.

## Figures and Tables

**Table 1:** Dopaminergic proteins expressed in immune cells.

	D1R	D2R	D3R	D4R	D5R	VAT1/2	DAT	TH	DDC	D*β*H	PAH
Cellular types	T cell [76]^§‡†^, [77]^§‡^B cells [77]^§‡^, [78]^§‡^Dendritic cells [75]^*∗*†^ HMDM [79]^§‡^, [80]^§‡†^, [81]^§‡†^ NK cells [82]^*∗*‡†^ CD4^+^ CD25^−^ T lymphocytes [83]^§†,↔^ Teff [84]^*∗*‡↔^ CD4^+^ lymphocytes (naive, central memory, effector memory) [85]^§†^ Treg [83]^§†,↔^, [84]^*∗*‡^ Tonsil [86]^§‡^ T cell mesenteric lymph nodes [87]^*∗*‡^	B cells [88]^§†^, [78]^§‡^T cell [76]^§‡†^, [88]^§†^, [89]^§‡†^, [90]^§†^ CD4^+^CD25^−^ T lymphocytes [83]^§†^ Teff [84]^*∗*‡^ CD4^+^ lymphocytes (naive, central memory, effector memory) [85]^§†^, [88]^§†^, [91]^§‡^ Treg [83]^§‡^, [84]^*∗*‡^ Dendritic cells [75]^*∗*†^ HMDM [79]^§‡^, [80]^§‡†^ Monocytes [88]^§†^ Eosinophils [88]^§†^ Neutrophils [88]^§†^ NK cell [88]^§†^, [82]^*∗*‡†^ Resting NK cells [92]^§‡†^ PBMC [93]^§‡^ T cell CD4^+^ resting [86]^§‡^ T cell CD4^+^ activated [86]^§‡^ Tonsil, thymus [86]^§‡^ T cell mesenteric lymph nodes [87]^*∗*‡^	B cells [88]^§†^ , [78]^§‡^Lymphocytes [94]^§†^, [95]^§‡^, [96]^§†^ CD4^+^ CD25^−^ T lymphocytes [83]^§‡^, [88]^§†^ Teff [84]^*∗*‡^ CD4^+^ lymphocytes (naive, central memory, effector memory) [85]^§†^ Treg [83]^§‡†^, [84]^*∗*‡^ T cells [97]^*∗*‡^, [89]^§‡†^ T cell [90]^§†^ Dendritic cells [75]^*∗*†^ HMDM [79]^§‡†^ Monocytes [88]^§†^ Eosinophils [88]^§†^ Neutrophils [88]^§†^ NK cell [88]^§†^, [82]^*∗*‡†^ Resting NK cells [92]^§‡†^ PBMC [93]^§‡^ T cell CD4^+^ resting [86]^§‡†↔^ T cell CD8^+^ resting [86]^§‡†^, [98]^§‡^ Tonsil, thymus, lymph nodes [86]^§‡^ T cell mesenteric lymph nodes [87]^*∗*‡^	B cells [88]^§†^ Lymphocytes [94]^§†^, [96]^§†^, [99]^§‡^CD4^+^ CD25^−^ T lymphocytes [83]^§†^ Teff [84]^*∗*‡^, [88]^§†^ CD4^+^ lymphocytes (naive, central memory, effector memory) [85]^§†^ Treg [83]^§‡^, [84]^*∗*‡^ Anti-CD3^+^CD28^+^ induced T cells [100]^§†^ HMDM [79]^§‡†^ Monocytes [88]^§†^ Eosinophils [88]^§†^ Neutrophils [88]^§†^ NK cell [88]^§†^, [82]^*∗*‡†^ Resting NK cells [92]^§‡†^ PBMC [93]^§‡^ T cell CD8^+^ resting [86]^§‡^, [98]^§‡^ T cell CD8^+^ activated [98]^§‡^ Monocytes CD14^+^ resting [86]^§‡^ B cell CD19^+^ resting [86]^§‡^ Tonsil, thymus [86]^§‡^ T cell mesenteric lymph nodes [87]^*∗*‡^	B cells [88]^§†^, [78]^§‡^ Lymphocytes [101]^§‡^, [102]^§‡†^CD4^+^ CD25^−^ T lymphocytes [83]^§†^, [88]^§†^ CD4^+^ lymphocytes (naive, central memory, effector memory) [85]^§†^ T cell [90]^§†^ Treg [83]^§‡†↔^, [84]^*∗*‡^ Dendritic cells [75]^*∗*†^ HMDM [79]^§‡^ Monocytes [88]^§†^ Eosinophils [88]^§†^ Neutrophils [88]^§†^ NK cell [88]^§†^, [82]^*∗*‡†^ Resting NK cells [92]^§‡†^ PBMC [93]^§‡†^ Thymus [86]^§‡^ T cell CD8^+^ resting [98]^§‡↔^ T cell mesenteric lymph nodes [87]^*∗*‡^	Immature dendritic cells [75]^*∗*‡^Lymphocytes [103]^§†^ CD4^+^ CD25^−^ T lymphocytes [83]^§‡^ HMDM [79]^§‡†^ Treg [83]^§‡^	Lymphocytes [103]^§†^, [104]^§†^, [105]^§†^, [91]^§‡^HMDM [79]^§‡†^ B cells [78]^§‡^	Lymphocytes [103]^§†^, [106]^§†^PBMC, T and B Lymphocytes [77]^§‡^ Treg [83]^§‡^ HMDM [79]^§‡†^ Th1 and Th2 Lymphocytes [107]^*∗*‡†^ Dendritic cells [75]^*∗*†^ Lymphocytes ConA activated [108]^*∗*‡^ Mesenteric lymph nodes, thymus, lymphocytes [108]^*∗*†^	HMDM (human monocyte-derived macrophages) [79]^§‡†^Leukocytes [109]^§‡†^ U937 cells, model of human macrophages [110]^§‡†^	T/B cells [111]^*∗*‡^	Leucocytes [112]^†^, [113]^†^, [114]^§†^

^§^Human, ^*∗*^murine, ^‡^expression, ^†^protein, and ^↔^very low signal.

**Table 2:** Dopaminergic system protein components expressed in immune system.

Receptors of DRs	Genes names	References
D(1) receptor: D1R^*∗*^ (old name D1A receptor) D1-like subtype	*DRD1* ^*∗*^	[75–87]
D(2) receptor longer: D2R D2-like subtype	*DRD2*	[75, 76, 78–80, 82–93]
D(3) receptor: D3R D2-like subtype	*DRD3*	[75, 78, 79, 82–90, 92–98]
D(4) receptor: D4R D2-like subtype	*DRD4*	[79, 82–88, 92–94, 96, 98–100]
D(5) receptor: D5R (old name D1B receptor) D1-like subtype	*DRD5*	[75, 78, 79, 82–88, 90, 92, 93, 98, 101, 102]
Sodium-dependent dopamine transporter: DAT	*SLC6A3* (synonyms: *DAT1*)	[78, 79, 91, 103–105, 115, 116]

Protein components shared with other monoamines systems		
Chromaffin granule amine transporter: VAT1	*SLC18A1* isoform 2 (synonyms: *VMAT1*)	[83, 103, 117, 118]
Synaptic vesicular amine transporter: VAT2	*SLC18A2* (synonyms: *VMAT2*)	[75, 79, 83, 103, 116]

Protein components with unknown functions		
Chromaffin granule amine transporter: VAT1Δ15^↔^	*SLC18A1* isoform 2 (synonyms: *VMAT1*Δ*15*)	[117, 118]
*Pseudogene* D(5) receptor^†^	*ψDRD5-1*	[101, 119–121]
*Pseudogene* D(5) receptor^†^	*ψDRD5-2*	[101, 119–121]

^*∗*^This gene expresses low levels of mRNA and translates low quantities of the protein.

^†^Unknown peptide product but produces mRNA with unknown functions.

^↔^This protein cannot transport serotonin.

**Table 3:** The DRs effect on cytokine production.

Effect on cytokine production	Cellular types and stimuli	Receptors involved	References
↑ IL-6^‡†^ ↑ CCL2^‡†^	HMDM + DA	D3R, D4R^*∗*^	[79]

↑ IL-6^‡^ ↑ CCL2^‡†^ ↑ CXCL8^‡†^ ↑ IL10^‡†^ ↓ TNF-*α* ^‡†^	HMDM + DA + LPS	D3R, D4R^*∗*^	[79]

↑ IFN-*γ* ^‡^ ↓ IL-10^‡^ ↓ IL-4^‡^	Human activated T cells + quinpirole	D3R	[97]

↑ IFN-*γ* ^‡^ ↓ IL-10^‡^ ↓ IL-4^‡^	Human activated CD4 T cells + quinpirole	D3R	[97]

↑ IFN-*γ* ^‡^	Human activated CD8 T cells + quinpirole	D3R	[97]

↑ IFN-*γ* ^‡^	Rats T cells + L-DOPA + carbidopa	D3R	[97]

↓ IL-12^†^ ↓ IL-23^‡^	D5RKO mice, mature bone marrow-derived dendritic cells + LPS	D5R absence	[75]

↓ IFN-*γ* ^†^ ↓ IL-2^†^ ↓ IL-4^†^	Anti-CD3-stimulated human T cells + DA Anti-CD3-stimulated human T cells + SCH-23390 Anti-CD3-stimulated human T cells + clozapine	Not measured D1R/D5R D4R	[89]

↓ IL-10^†‡^ ↓ TGF-*β* ^†‡^	Human CD4^+^ CD25^+^ regulatory T cells: (a) + reserpine + L-741626 (b) + reserpine + U-99194A (c) + reserpine + L-741741	(a) D2R, D3R, D4R (b) D3R (c) D4R	[83]

↓ IL-2^†^ ↓ IFN-*γ* ^†^ ↓ IL-6^†^ ↓ IL-2^†^ ↓ IFN-*γ* ^†^ ↓ IL-6^†^	Mouse activated lymphocytes + L-DOPA Mouse activated lymphocytes + L-DOPA Mouse activated lymphocytes + L-DOPA Mouse activated lymphocytes + DA Mouse activated lymphocytes + DA Mouse activated lymphocytes + DA	Not measured	[122]

↑ IL-4^†^ ↑ IL-5^†^	Naive CD4^+^ T cells + DA	D1R/D5R	[123]

↓ IFN-*γ* ^†^	Human resting NK cells + DA	D5R	[92]

↑ IFN-*γ* ^‡†^ ↓ IFN-*γ* ^‡^	Human rIL-2 activated NK cells + DA Human rIL-2 activated NK cells + SKF-38393	D5R D5R	[92]

↑ IL-17^†^ ↓ IFN-*γ* ^†^ ↓ IL-17^†^ ↑ IFN-*γ* ^†^	Immature human Mo-Dc + L75066 Immature human Mo-Dc + L75066 Immature human Mo-Dc + SHC23390 Immature human Mo-Dc + SHC23390	D2-*like* D2-*like* D1R/D5R D1R/D5R	[124]

↑ IL-10^‡†^ ↑ TNF-*α* ^‡†^	T cells + DA T cells + DA	D2R, D1-*like* D3R, D1-*like*	[90]

↓ IL-2^†^	Anti-CD3/CD28 stimulated T lymphocytes + PD168,077	D4R	[100]

↓ IL-2^†^ ↓ IL-10^†^	Anti-CD3/CD28 activated Treg lymphocytes + DA Anti-CD3 + IL-2 activated Treg lymphocytes + DA	Not measured	[84]

↓ IL-2^‡†^	Anti-CD3/CD28 activated T lymphocytes CD8^+^ + DA	Not measured	[98]

↓ IFN-*γ* ^†^ ↓ IL4-*γ* ^†^	Lymphocytes + DA	Not measured	[20, 125]

↓ IFN-*γ* ^†^ ↓ IFN-*γ* ^†^ ↑ IL-4-*γ* ^†^	T cell (mesenteric lymph nodes) + ConA + SKF38393 T cell (mesenteric lymph nodes) + ConA + quinpirole T cell (mesenteric lymph nodes) + ConA + quinpirole	D1-*like* D2-*like* D2-*like*	[87]

↑ TNF-*α* ^†^ ↑ IL-10^†^ ↓ IFN-*γ* ^†^ ↓ IL-10^†^ ↑ IgG^†^	Macrophages DAT^−/−^ mice + LPS Macrophages DAT^−/−^ mice + LPS Splenocytes DAT^−/−^ mice activated Splenocytes DAT^−/−^ mice activated Serum DAT^−/−^ mice	DAT absence	[126]

^‡^Expression.

^†^Protein.

^*∗*^D3R and D4R were only detected by western blot.

**Table 4:** Pathology-associated dopaminergic protein expression in immune cells.

Pathology/condition	DA receptors and cell types involved	Reference
Parkinson disease Patients/MPTP mice	↓ D3R in lymphocytes	[127]

Lesch-Nyhan disease	↑ D5R in lymphocytes	[128]

Multiple sclerosis	↓ D5R in lymphocytes	[129]

Multiple sclerosis treatment with IFN-*β*	↑ D5R in lymphocytes ↓ D2R in lymphocytes	[129, 130]

Schizophrenia	↑ D4R in T CD8^+^ and T CD4^+^ lymphocytes ↑ D4R in T CD8^+^ lymphocytes ↓ D2R in T CD4^+^ lymphocytes	[131]

Rheumatoid arthritis type II collagen-induced arthritis (mice)	↑ D2R in lymphocytes	[132]

Rheumatoid arthritis and osteoarthritis	↓ D5R in B cells ↑ D2R & D3R in B cells	[133]

Systemic lupus erythematosus	↑ D4R in lymphocytes ↓ D2R in lymphocytes	[134]

Alcohol dependence syndrome	↑ D4R in lymphocytes	[135]

Alcohol withdrawal	↑ D1R in lymphocytes	[136]

Computer game addicts	↓ D5R in lymphocytes	[137]
